# 5-Methylcytidine RNA Epitranscriptomics in Women’s Health and Disease: Mechanisms and Clinical Implications

**DOI:** 10.3390/cells15090847

**Published:** 2026-05-06

**Authors:** Qiwei Yang, Sana M. Salih, Rongxue Wu, Itika Arora, Mira Mousa, Ayman Al-Hendy, Thomas G. Boyer

**Affiliations:** 1 Department of Obstetrics and Gynecology, University of Chicago, Chicago, IL 60637, USA; aalhendy@bsd.uchicago.edu; 2 Division of Reproductive Endocrinology and Infertility, Department of Obstetrics and Gynecology, University of Chicago, Chicago, IL 60637, USA; sana.salih@bsd.uchicago.edu; 3 Section of Cardiology, Department of Medicine, Biological Sciences Division, University of Chicago, Chicago, IL 60637, USA; 4 Division of Cardiac Surgery, Department of Surgery, Dorothy M. Davis Heart & Lung Research Institute, Ohio State University, Columbus, OH 43210, USA; wu170@osumc.edu; 5 Department of Public Health and Epidemiology, Khalifa University, Abu Dhabi P.O. Box 127788, United Arab Emirates; itika.arora@ku.ac.ae (I.A.); mira.imousa@ku.ac.ae (M.M.); 6 Center for Biotechnology, Khalifa University, Abu Dhabi P.O. Box 127788, United Arab Emirates; 7 Department of Medical Sciences, Khalifa University, Abu Dhabi P.O. Box 127788, United Arab Emirates; 8 Department of Obstetrics and Gynecology, Sheik Shakhbout Medical City, Abu Dhabi P.O. Box 127788, United Arab Emirates; 9 Department of Molecular Medicine, Institute of Biotechnology, University of Texas Health Science Center at San Antonio, San Antonio, TX 78229, USA; boyer@uthscsa.edu

**Keywords:** 5-methylcytidine (m^5^C), RNA modification, epitranscriptomics, women’s health, hormone-responsive tissues, reproductive biology, gynecologic diseases, placenta and pregnancy, metabolic homeostasis, vascular disease, cardiac remodeling, biomarkers and therapeutics

## Abstract

**Highlights:**

**What are the main findings?**

- 5-methylcytidine (m^5^C) is a dynamic RNA modification that regulates RNA stability, translation, localization, and stress responses across diverse biological systems.
- m^5^C dysregulation is implicated in key processes relevant to women’s health, including early embryonic development, reproductive function, pregnancy adaptation, metabolic regulation, and cancer progression.

**What are the implications of the main findings?**

- m^5^C represents a critical epitranscriptomic regulatory layer linking RNA metabolism with hormone-responsive physiology and sex-specific disease mechanisms.
- It has strong potential as a biomarker and therapeutic target, supporting future precision medicine approaches in women’s health and disease.

**Abstract:**

Chemical modifications of RNA add a dynamic regulatory layer to gene expression beyond the genome and epigenome. Among these modifications, 5-methylcytidine (m^5^C) has emerged as a key epitranscriptomic modification that influences RNA stability, translation, localization, and stress responses across diverse biological systems. Recent advances in high-resolution mapping and functional interrogation of m^5^C have revealed its involvement in development, metabolism, immune regulation, and disease pathogenesis. Notably, many of these processes are highly relevant to women’s health, which is shaped by hormone-responsive tissues, reproductive transitions, and pregnancy-associated physiological adaptations. In this review, we provide a comprehensive and integrative overview of m^5^C RNA modification with a focus on its roles in female biology and disease. We summarize the molecular machinery responsible for m^5^C deposition, recognition, and regulation, as well as current detection technologies. We further highlight emerging evidence linking m^5^C dysregulation to early embryonic development, women-specific cancers, gynecologic and reproductive disorders, pregnancy complications, and metabolic and cardiovascular diseases. In addition, we discuss the interplay between m^5^C and sex hormone signaling, as well as the potential of m^5^C as a biomarker and therapeutic target. Finally, we identify key knowledge gaps, including the need for tissue-specific, longitudinal, single-cell, and spatial epitranscriptomic studies in women. By integrating epitranscriptomics into women’s health research, this review underscores m^5^C as a previously underappreciated regulatory layer with significant implications for precision medicine and clinical translation.

## 1. Introduction

### 1.1. Epigenetics vs. Epitranscriptomics: Expanding Regulatory Layers

Epigenetic regulation has long been recognized as a fundamental mechanism controlling gene expression without altering the underlying DNA sequence. It operates primarily through DNA methylation, post-translational histone modifications (such as acetylation, methylation, phosphorylation, and ubiquitination), and ATP-dependent chromatin remodeling complexes. These processes regulate chromatin accessibility and transcription factor binding, thereby shaping gene expression landscapes in a cell-type–specific manner [1]. Through the coordinated deposition and removal of epigenetic marks, cells establish relatively stable transcriptional programs that define cellular identity, guide lineage commitment during development, and maintain long-term transcriptional memory across cell divisions. Dysregulation of these mechanisms contributes to developmental disorders, aging, and a wide range of diseases, including reproductive disorders, cancer and cardiovascular pathology [2,3].

In contrast, epitranscriptomics represents a more recently appreciated layer of gene regulation that functions at the RNA level [4]. Chemical modifications of RNA, including m^6^A, pseudouridine and m^5^C, are installed, interpreted, and removed by specificalized proteins commonly referred to as writer, reader, and eraser proteins [5]. Unlike many epigenetic marks, RNA modifications are highly dynamic and can rapidly adjust in response to intracellular signaling and environmental stimuli. By modulating RNA structure and interactions with RNA-binding proteins, epitranscriptomic marks influence virtually every stage of RNA metabolism, including splicing, nuclear export, subcellular localization, translation efficiency, and transcript stability or decay [6]. This regulatory flexibility enables rapid and context-dependent fine-tuning of gene expression at the post transcription level, providing cells with a flexible and reversible mechanism to rapidly adapt protein production to developmental cues, hormonal fluctuations, metabolic demands, and stress conditions [7].

Together, epigenetic and epitranscriptomic mechanisms form an integrated, multilayered regulatory network that coordinates gene expression across DNA, chromatin, and RNA levels. Epigenetic programs establish transcriptional potential, while epitranscriptomic modifications refine and adjust transcript utilization and protein synthesis. This interplay greatly expands the complexity, precision, and plasticity of gene regulation beyond transcription alone. Understanding how these layers interact provides deeper insight into tissue-specific physiology, sex differences, adaptive stress responses, and disease pathogenesis. It opens new avenues for therapeutic intervention targeting regulatory processes at both chromatin and RNA levels.

### 1.2. Overview of RNA Modifications with Focus on m^5^C

Post-transcriptional chemical modification of RNA adds a critical regulatory layer to gene expression, collectively referred to as the epitranscriptome [8,9]. To date, more than 170 distinct RNA modifications have been identified across diverse RNA species, including transfer RNA (tRNA), ribosomal RNA (rRNA), messenger RNA (mRNA), and various classes of noncoding RNA. These modifications influence nearly every aspect of RNA metabolism, including folding, stability, splicing, nuclear export, translation efficiency, and decay. Among the best-characterized RNA modifications are N6-methyladenosine (m^6^A), pseudouridine (Ψ), 5-methylcytidine (m^5^C), N1-methyladenosine (m^1^A), and 2′-O-methylation, each of which contributes uniquely to post-transcriptional gene regulation [5,6]. However, the dynamic regulation and physiological relevance of RNA m^5^C installation and removal mechanisms remain incompletely defined, and in some contexts, their roles are still debated.

m^5^C was originally identified in highly abundant and structurally conserved RNAs, particularly tRNA and rRNA, where it plays essential roles in maintaining RNA stability, proper folding, and translational fidelity [10,11]. In tRNA, m^5^C modifications enhance structural integrity [11,12] and protect transcripts from stress-induced cleavage [13], whereas in rRNA they contribute to ribosome biogenesis and function [14,15]. Moreover, transcriptome-wide profiling approaches have revealed that m^5^C is also present in mRNA and long noncoding RNAs [16,17,18], expanding its functional relevance beyond housekeeping processes to the dynamic regulation of gene expression [19,20,21].

The installation of m^5^C is catalyzed primarily by S-adenosylmethionine (SAM)–dependent RNA methyltransferases of the NOP2/Sun RNA methyltransferase (NSUN) family (NSUN1–7) and DNMT2 (DNA Methyltransferase 2) [also known as TRDMT1 (tRNA aspartic acid methyltransferase 1)] [12,22,23]. These enzymes exhibit distinct RNA substrate specificity and tissue-dependent expression patterns, enabling context-dependent regulation of m^5^C deposition. In contrast to DNA 5-methylcytosine, which is often associated with transcriptional repression and chromatin compaction [24], RNA m^5^C modification influences multiple steps of RNA metabolism, including RNA nuclear export, transcript stability, translational efficiency, and stress-responsive gene regulation [25]. Rather than serving as a static epigenetic mark, RNA m^5^C acts as a dynamic and context-dependent regulator that fine-tunes gene expression. Emerging evidence further suggests that RNA m^5^C is reversibly regulated by members of the TET and AlkB homolog families, supporting a potential demethylation pathway [26]. In addition, m^5^C-modified transcripts can be selectively recognized by reader proteins, such as Aly/REF export factor (ALYREF) and YBX1, which mediate RNA nuclear export, stability, and translational control [18,27,28].

Compared with m^6^A, the most extensively studied mRNA modification, m^5^C appears to be potentially more stable, with erasure mechanisms remaining incompletely defined. This relative stability suggests that m^5^C may serve as a longer-term regulatory signal that integrates developmental cues, cellular identity, and environmental responses. Notably, m^5^C frequently coexists and functionally interacts with other RNA modifications, highlighting extensive crosstalk within the epitranscriptomic landscape [29]. Such multilayered regulation enables fine-tuning of gene expression programs in a cell-type- and condition-specific manner.

Together, these objectives provide a conceptual framework for understanding how m^5^C-mediated epitranscriptomic regulation shapes women’s health and establish a foundation for future mechanistic and translational studies [30].

### 1.3. Rationale for Focusing on Women’s Health

Women’s health provides a unique and biologically compelling context in which to investigate epitranscriptomic regulation. Many tissues central to women’s physiology are highly dynamic and sensitive to hormonal, developmental, and environmental cues, requiring precise and adaptable control of gene expression. RNA-based regulatory mechanisms are particularly well-suited to meet these demands, yet their contributions to female-specific biological processes and diseases remain underexplored.

Hormone-responsive tissues such as the breast, uterus, ovary, and endometrium, undergo cyclical and life-stage–dependent remodeling driven largely by estrogen and progesterone signaling. Across the female life course, from menstrual cycling through pregnancy, lactation, and menopause, these tissues undergo repeated and coordinated transition in gene expression that regulate cellular proliferation, differentiation, and tissue architecture [31]. Epitranscriptomic modifications such as m^5^C provide a mechanistic framework for fine-tuned regulation by modulating RNA stability, localization, and translation in response to hormonal signals. Understanding how m^5^C machinery interfaces with hormone receptor pathways may therefore reveal new layers of regulation underlying both normal physiology and hormone-dependent pathologies.

Reproductive biology further underscores the relevance of RNA modifications in women’s health. Oogenesis, follicular development, fertilization, and early embryogenesis rely heavily on post-transcriptional regulation, as transcriptional activity is temporally restricted during key stages. RNA modifications play critical roles in ensuring the stability and translation competence of maternal transcripts during these windows [32,33]. Given its established functions in RNA integrity and translational control [34], m^5^C is well-positioned to influence reproductive competence, fertility, and pregnancy outcomes. Yet, its specific contributions in female reproductive tissues remain incompletely defined.

Finally, many diseases exhibit pronounced sex-biased prevalence, presentation, and outcomes, including autoimmune disorders, metabolic syndromes, cardiovascular disease, and hormone-dependent cancers [35,36,37,38,39]. Gynecologic conditions such as endometriosis [40], uterine fibroids (UFs) [41], and ovarian, endometrial, and uterine cancers [42,43,44] affect women and often involve dysregulated gene expression, inflammation, and tissue remodeling. Investigating m^5^C in these contexts may help explain sex-specific molecular vulnerabilities and identify novel biomarkers or therapeutic targets tailored to women’s health. Together, these considerations highlight the importance of centering women’s biology in epitranscriptomic research and position m^5^C as a promising regulatory mechanism at the intersection of hormones, reproduction, and disease susceptibility.

### 1.4. Scope and Objectives of the Review

The scope of this review is to provide a comprehensive and integrative overview of m^5^C RNA modification as an emerging regulatory mechanism in women’s health and disease. We focus on current understanding of the molecular machinery governing m^5^C deposition, recognition, and function, with particular emphasis on its roles in hormone-responsive tissues, reproductive biology, and female-specific physiological processes across the life course. By synthesizing findings from studies of diverse RNA species, including tRNA, rRNA, mRNA, and long noncoding RNA, this review highlights how m^5^C contributes to post-transcriptional gene regulation in contexts relevant to women’s biology.

The objectives of this review are fourfold. First, we aim to summarize the biochemical and mechanistic foundations of m^5^C regulation, including key writers, readers, and emerging detection methodologies, while distinguishing RNA m^5^C from DNA 5-methylcytosine–mediated epigenetic control. Second, we examine the physiological roles of m^5^C in female development, reproductive function, and pregnancy, emphasizing life-stage– and tissue-specific regulation. Third, we critically evaluate evidence linking dysregulation of m^5^C machinery to women-predominant disorders, including gynecologic diseases, infertility, and hormone-dependent cancers. Finally, we discuss the translational potential of m^5^C as a source of biomarkers and therapeutic targets, identify current knowledge gaps, and outline key directions for future research.

By defining these boundaries and goals, this review seeks to provide a conceptual framework for understanding how m^5^C-mediated epitranscriptomic regulation shapes women’s health and to offer a roadmap for future mechanistic and translational studies in this rapidly evolving field.

## 2. Molecular Basis of m^5^C RNA Modification

**Figure 1:**
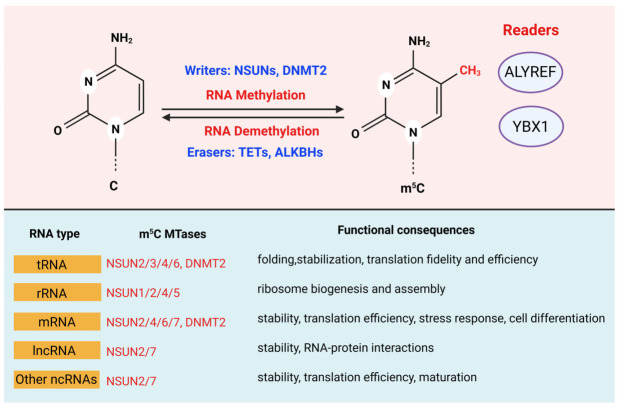
**Overview of RNA m^5^C modification, associated enzymes, and functional consequences. Upper panel:** Schematic representation of m^5^C RNA modification and its regulatory machinery. RNA cytosine residues are methylated by m^5^C “writer” enzymes, including members of the NSUN family and DNMT2, resulting in the formation of m^5^C. m^5^C-modified RNAs are recognized by reader proteins such as ALYREF and YBX1, which mediate downstream effects on RNA metabolism. **Lower panel**: summary of major RNA substrates of m^5^C modification, the corresponding methyltransferases, and their functional consequences. m^5^C modification of tRNA regulates folding, stabilization, and translation fidelity; rRNA modification controls ribosome biogenesis and assembly; mRNA methylation influences stability, translation efficiency, stress responses, and cell differentiation; lncRNA methylation modulates RNA biogenesis, processing, stability, and RNA–protein interactions; and other noncoding RNAs are involved in transcription elongation and RNA splicing. MTases: methyltransferases.

m^5^C is an evolutionary conserved post-transcriptional RNA modification formed by the covalent addition of a methyl group to the carbon-5 position of cytidine [45]. This modification occurs across multiple RNA classes, including tRNA, rRNA, mRNA, and lncRNA. It influences RNA structure and function at multiple levels (Figure 1). Unlike DNA 5-methylcytosine, which primarily regulates transcription through chromatin-based mechanisms, m^5^C modulates gene expression predominantly at the post-transcriptional level, affecting RNA stability, localization, and translation.

### 2.1. Chemical Nature and Distribution of m^5^C

At the chemical level, m^5^C alters base-stacking interactions and RNA secondary structure without disrupting canonical Watson–Crick base pairing. This subtle structural modification enhances RNA stability and modulates the accessibility of RNA-binding proteins and ribonucleoprotein complexes.

m^5^C is abundant in tRNA and rRNA, where it occupies evolutionarily conserved positions critical for RNA folding and translational fidelity. In contrast, m^5^C sites in mRNA and lncRNA are less frequent but strategically enriched within coding sequences and untranslated regions (UTR), suggesting roles in regulating transcript stability, localization, and translation efficiency. Transcriptome-wide mapping studies have further revealed that m^5^C deposition can be cell-type-specific and dynamically regulated across developmental and physiological conditions. These findings indicate that m^5^C not only contributes to structural RNA integrity but also participates in adaptive gene regulatory programs. Notably, although m^5^C is enriched at specific transcript regions, the determinants governing site selectivity and functional outcomes remain incompletely understood.

### 2.2. m^5^C Writers: RNA Cytosine Methyltransferases

The installation of RNA m^5^C is catalyzed by SAM–dependent cytosine methyltransferases, primarily members of the NSUN RNA methyltransferase family and DNMT2, which collectively regulate RNA stability, splicing, nuclear export, and translation [46,47]. The NSUN family comprises NSUN1–7, enzymes that share a conserved catalytic cysteine residue that forms a covalent intermediate with the C6 position of cytosine, thereby activating the C5 carbon for methyl group transfer from SAM to generate m^5^C [48,49]. Individual NSUN proteins display distinct substrate specificities and subcellular localizations: NSUN1 and NSUN5 predominantly modify rRNA; NSUN2 targets tRNA and selected mRNAs; NSUN3 methylates mitochondrial tRNA; and NSUN6 modifies specific cytoplasmic tRNAs, enabling cell type– and context-dependent regulation of m^5^C deposition [26]. DNMT2 was initially classified as a DNA methyltransferase but is now recognized as primarily methylating tRNA substrates. DNMT2 contributes to tRNA stability and protects transcripts from stress-induced cleavage, thereby supporting translational homeostasis (Figure 1). Emerging evidence suggests that m^5^C writers may also function within broader epitranscriptomic networks, interacting with other RNA modification systems. For example, coordinated modification of shared transcripts by m^5^C writers and the METTL3/METTL14 complex, which installs m^6^A has been observed in transcripts (e.g., p21 mRNA), indicating potential cross-talk between different RNA modification pathways. Collectively, these m^5^C methyltransferases form a multilayered regulatory network that not only sustains fundamental cellular processes but also contributes to pathological states, including tumor initiation, progression, and adaptation within the tumor microenvironment [50]. However, how substrate specificity is achieved in vivo and how different NSUN enzymes coordinate across tissues remain key unresolved questions.

### 2.3. m^5^C Readers: Recognition and Functional Interpretation

The biological functions of RNA m^5^C are largely mediated by reader proteins that selectively recognize methylated transcripts and translate this epitranscriptomic mark into functional outcomes. Key m^5^C readers include ALYREF, Y-Box Binding Protein 1 (YBX1), and YTH N6-Methyladenosine RNA Binding Protein F2 (YTHDF2), which regulate RNA stability, nuclear export, translation, and degradation. ALYREF, the first identified m^5^C reader, recognizes methylated RNA via Lys171 and functions within the transcription–export complex (TREX) to facilitate nuclear export of m^5^C-modified transcripts, thereby linking methylation to RNA trafficking, splicing, and translational efficiency [18,51,52,53]. YBX1 binds m^5^C-modified RNA through the Trp45 residue in its cold-shock domain, enhancing transcript stability and promoting processes such as proliferation, migration, and drug resistance in cancer cells [54,55]. YTHDF2, originally characterized as an m^6^A reader, has also been reported to recognize m^5^C-modified RNA under specific conditions. Importantly, the specificity of m^5^C recognition by canonical m^6^A readers such as YTHDF2 remains an area of active investigation and debate. Notably, m^5^C recognition by YTHDF2 requires cooperative engagement of multiple key amino acids, suggesting distinct structural requirements compared with m^6^A binding [26,56]. Collectively, these reader proteins determine the fate of methylated RNAs and contribute to the dynamic regulation of gene expression in development, stress responses, and tumorigenesis, underscoring the importance of m^5^C recognition in epitranscriptomic control [26,50,53,57].

### 2.4. m^5^C Erasers and Modification Dynamics

Although m^5^C in RNA was initially considered a relatively stable modification, accumulating evidence suggests that it may be dynamically regulated in specific contexts. Proposed m^5^C “erasers” include members of the Ten-Eleven Translocation (TET) family and AlkB homolog proteins; however, their roles in RNA demethylation remain incompletely established and, in some cases, controversial [26,58,59]. Through demethylation or oxidative modification, these enzymes are implicated in controlling RNA stability, post-transcriptional processing, translation efficiency, and gene expression, thereby influencing cell differentiation, stress responses, and tumorigenesis [19,30].

Among these, TET family enzymes are the most extensively studied. Analogous to their role in DNA demethylation [60], TET proteins catalyze Fe^2+^- and α-ketoglutarate–dependent oxidation of m^5^C, generating 5-hydroxymethylcytosine (hm^5^C), 5-formylcytosine (f^5^C), and 5-carboxylcytosine (ca^5^C). These oxidized intermediates can subsequently be repaired or removed, ultimately restoring unmodified cytosine. This stepwise oxidation mechanism provides a potential pathway for reversible m^5^C regulation in RNA [61]. In contrast, members of the AlkB homolog family (ALKBH) employ a distinct catalytic strategy. While also dependent on Fe^2+^ and α-ketoglutarate, ALKBH enzymes are proposed to catalyze direct demethylation without generating stable oxidative intermediates, thereby removing the methyl group from m^5^C and regenerating cytosine via a distinct biochemical route [62]. Collectively, these findings indicate that although both TET and ALKBH enzymes are Fe^2+^/alpha-ketoglutarate (α-KG)-dependent dioxygenases, ALKBH proteins employ a distinct biochemical route characterized by direct oxidative demethylation rather than by stepwise generation of stable oxidation intermediates, providing an alternative potential mechanism for the reversible regulation of m^5^C in RNA [26,50,57].

## 3. Methodologies for Detecting m^5^C

Accurate detection and quantification of m^5^C are essential for elucidating its biological functions and disease relevance. Unlike DNA m^5^C, m^5^C in RNA presents unique technical challenges due to RNA secondary structure, lower abundance, and the chemical similarity of m^5^C to unmodified cytidine. Multiple complementary approaches have therefore been developed to map m^5^C at varying resolutions, each with distinct advantages and limitations.

### 3.1. Mass Spectrometry

Mass spectrometry (MS), particularly LC–MS/MS, is a gold-standard method for detecting RNA m^5^C due to its high sensitivity, specificity, and quantitative accuracy. MS operates by measuring the mass-to-charge (*m*/*z*) ratios of ionized molecules, enabling precise determination of molecular weight and chemical composition. In the context of RNA modification analysis, RNA is enzymatically digested into individual nucleosides, which are then separated by liquid chromatography and subsequently identified and quantified by tandem mass spectrometry based on their unique fragmentation patterns. This approach allows accurate detection of modified nucleosides, including m^5^C, with excellent chemical resolution. LC–MS/MS is particularly powerful because it enables simultaneous detection and quantification of multiple RNA modifications within a single analytical run. This multiplexing capability makes it highly suitable for global epitranscriptomic profiling and comparative studies across different biological conditions, tissues, or disease states [63]. These characteristics collectively contribute to its high reproducibility and chemical definitiveness in validating both the presence and relative abundance of m^5^C in RNA samples.

Despite its chemical precision, mass spectrometry cannot typically provide transcript-specific or single-nucleotide positional information because RNA must be enzymatically hydrolyzed into nucleosides before analysis, thereby losing sequence context. As a result, MS measures global or RNA-class–specific m^5^C abundance rather than mapping exact modification sites. Furthermore, the method requires specialized instrumentation, technical expertise, and relatively large amounts of input RNA, which may limit its application to low-input or rare cell populations. Sample preparation can also introduce variability and distinguishing isomeric or closely related modifications may require advanced fragmentation strategies. Thus, while MS is highly accurate for quantification, it is not suitable for transcriptome-wide site mapping without complementary approaches [25].

### 3.2. Antibody-Based Methods

Antibody-based methods for detecting RNA m^5^C, such as m^5^C-RNA immunoprecipitation sequencing (m^5^C-RIP-seq), miCLIP, offer several important advantages for epitranscriptomic profiling. These approaches enable transcriptome-wide enrichment of m^5^C-containing RNAs without requiring harsh chemical treatments, thereby preserving RNA integrity compared with bisulfite-based methods. They are experimentally straightforward, compatible with standard next-generation sequencing workflows, and adaptable to diverse RNA classes, allowing global mapping of modification patterns across biological conditions. In addition, crosslinking-based adaptations such as miCLIP can achieve near single-nucleotide resolution, improving site specificity while maintaining the benefits of enrichment-based detection. Together, these features make antibody-based approaches scalable, relatively sensitive, and broadly applicable for identifying m^5^C landscapes in cells and tissues [64,65].

However, antibody-based detection methods for detecting RNA m^5^C have several notable limitations. First, they are highly dependent on antibody specificity and affinity; cross-reactivity or low specificity can generate false-positive enrichment signals and poor reproducibility across studies. Second, standard RIP-seq approaches provide relatively low resolution, typically identifying broad enriched regions (~100–200 nt) rather than precise single-nucleotide sites. Third, enrichment-based methods are generally semi-quantitative and do not accurately measure modification stoichiometry. In addition, miCLIP and Aza-IP detect only methyltransferase-specific m^5^C sites and require enzyme overexpression, limiting use to cultured cells and potentially perturbing the endogenous m5 C methylome. These technical constraints can lead to inconsistent m^5^C maps between datasets and limit confident site validation [66,67].

### 3.3. Bisulfite Sequencing–Based Approaches

RNA bisulfite sequencing (RNA-BS-Seq) adapts classical DNA bisulfite chemistry to RNA, exploiting the resistance of m^5^C to bisulfite-mediated deamination. During treatment, unmodified cytidines are converted to uridines, whereas m^5^C remains unchanged, allowing site-specific detection following reverse transcription and sequencing. RNA-BS-seq offers several significant advantages for detecting m^5^C in RNA. Most notably, it provides single-nucleotide resolution, enabling precise identification of modified cytosines within specific transcripts. Unlike antibody-based enrichment approaches, RNA-BS-seq is antibody-independent, thereby avoiding issues related to antibody specificity, cross-reactivity, and batch variability. The method also allows for quantitative estimation of methylation levels, as the proportion of cytosine retention at a given site reflects the degree of modification. When coupled with high-throughput sequencing, RNA-BS-seq enables transcriptome-wide analysis, allowing comprehensive mapping of m^5^C across coding and non-coding RNAs. Furthermore, the underlying bisulfite chemistry is well established and extensively validated in epigenetics research, making the approach technically accessible and adaptable for epitranscriptomic studies [18,66,68,69].

Despite its strengths, RNA-BS-seq has several important limitations. The sodium bisulfite treatment required for cytosine conversion is chemically harsh and often causes substantial RNA degradation, thereby reducing library complexity and transcript coverage. In addition, RNA secondary structures can hinder bisulfite accessibility, resulting in incomplete cytosine conversion and potentially false-positive identification of m^5^C sites. Even minor inefficiencies in conversion rates may significantly inflate background noise, necessitating rigorous controls and high sequencing depth. Furthermore, RNA-BS-seq cannot reliably distinguish m^5^C from other cytosine modifications that may also resist bisulfite conversion. The method also requires specialized bioinformatic pipelines and stringent filtering criteria to interpret conversion data accurately. Collectively, these technical challenges underscore the need for complementary validation approaches, such as methyltransferase knockout or orthogonal biochemical assays, to ensure confident m^5^C site identification [12,68,70].

### 3.4. Bisulfite-Free, TET-Assisted Chemical Oxidation Sequencing Method for Base-Resolution m^5^C Detection (m^5^C-TAC-seq)

m^5^C-TAC-seq is a bisulfite-free, nano base-resolution sequencing approach for detecting RNA m^5^C. The method uses TET enzyme–mediated oxidation to convert m^5^C into f^5^C, followed by selective chemical labeling and enrichment of oxidized sites. During reverse transcription and sequencing, modified positions generate characteristic C-to-T transitions, enabling direct identification of m^5^C at single-nucleotide resolution. Because it avoids bisulfite treatment, the method preserves RNA integrity and sequence complexity while allowing transcriptome-wide profiling and functional interrogation of methyltransferases (e.g., NSUN family enzymes). Despite its strengths, m^5^C-TAC-seq has limitations. The TET-mediated m^5^C-to-f^5^C oxidation method is ~50% efficient, so a biotin pull-down is required for sensitivity, limiting m^5^C-TAC-seq to semi-quantitative analysis and a ≥100 ng RNA input. Variable C-to-T conversion across RNA structures restricts absolute quantification, and further optimization is needed for low-input or single-cell use. Additionally, the method cannot distinguish m^5^C from hm^5^C, requiring refinement in contexts with higher hm^5^C levels [70].

### 3.5. Nanopore-Based Sequencing for Direct Detection of m^5^C

In addition to conventional approaches for detecting m^5^C, nanopore-based sequencing technologies have emerged as a powerful alternative for direct RNA modification analysis. Platforms developed by Oxford Nanopore Technologies enable real-time, single-molecule sequencing without the need for reverse transcription or amplification, thereby preserving native RNA modifications [71]. Recent advances in computational tools, such as Dorado, have further enhanced the capability of nanopore sequencing to accurately detect m^5^C modifications in RNA molecules. These algorithms leverage characteristic alterations in ionic current signals generated during RNA translocation through nanopores to distinguish modified from unmodified nucleotides [72].

Together, nanopore sequencing combined with improved basecalling models offers a promising, high-resolution approach for transcriptome-wide identification of m^5^C, expanding the toolkit for studying RNA epigenetic regulation.

## 4. m^5^C RNA Modification in Women’s Health: Physiological and Disease Contexts

Epitranscriptomic regulation, including m^5^C, represents an important layer of post-transcriptional gene control in female reproductive biology. While much of the mechanistic literature has historically focused on N^6^-methyladenosine (m^6^A), accumulating evidence indicates that m^5^C contributes to ovarian development, early embryogenesis, placental biology, hormone-responsive signaling, and reproductive disorders. Although mechanistic depth varies across systems, current findings collectively support a central role for m^5^C in maintaining reproductive homeostasis and mediating disease susceptibility.

### 4.1. Physiological Roles of 5-methylcytosine in Ovarian Development, Oogenesis, Folliculogenesis, and Ovulation

Ovarian oogenesis and folliculogenesis depend on tightly coordinated regulation of maternal transcript stability and translation within both oocytes and surrounding granulosa cells. Post-transcriptional regulation ensures proper timing of granulosa cell proliferation, steroidogenesis, and oocyte support. Although detailed mechanistic studies have more extensively characterized m^6^A [32], broader epitranscriptomic analyses recognize that m^5^C plays a role in the regulatory networks governing follicular development and reproductive competence [32,33]. Consistent with this, epitranscriptomic profiling across mammalian oocytes has revealed dynamic enrichment of RNA modifications, with relatively high m^6^A levels and lower but functionally significant m^5^C levels, coordinated through spatial localization of RNA-modifying enzymes to regulate maternal mRNA stability and translational timing during folliculogenesis and the early embryonic transition [73].

Ovarian folliculogenesis is a delicate and intricate process involving multiple steps throughout life. In females, it begins with oogenesis, which includes primordial germ cell (PGC) formation, specification, migration, proliferation, localization, and the development of primordial follicles (PFs). PGCs originate from the yolk sac and migrate to the genital ridges, where they merge with somatic cells to form undifferentiated gonads. The absence of the Y chromosome and the SRY gene, along with the expression of WNT4 genes in these gonads, results in a female phenotype (ovary). The oogonia (oocytes) are surrounded by a layer of somatic cells (pre-granulosa cells) to form primordial follicles [74]. Primordial follicles in the ovary develop in the cortex of the undifferentiated gonads [75]. The oocytes initiate meiosis, undergo epigenetic reprogramming, and experience X-chromosome inactivation or activation within the fetal ovary, entering prophase I, where homologous chromosomes pair, synapse, and exchange genetic material [76]. A newborn ovary contains only a fraction of the total oocytes that entered meiosis during fetal development. The oocyte remains arrested in the diplotene stage of prophase I until puberty, when folliculogenesis begins. Folliculogenesis involves cyclic recruitment and growth of follicles, marked by nuclear and cytoplasmic maturation that led to oocyte competence and ovulation under the regulation of pituitary and hypothalamic gonadotropin hormones. At puberty, meiosis I (M1) is triggered by an LH surge and results in germinal vesicle breakdown and chromosomal condensation, with the oocyte undergoing an asymmetric cytoplasmic division to produce secondary oocytes arrested at metaphase II and the first polar body [77]. Meiosis II (M2) occurs at fertilization, producing mature oocytes and the second polar body. Hormonal regulation of the ovarian cycle prompts the release of one dominant follicle each month. Several disease processes can interfere with this intricate sequence, leading to reproductive issues and infertility.

Emerging mechanistic studies further highlight that m^5^C modification serves as a critical regulator of maternal mRNA fate during folliculogenesis and the maternal-to-zygotic transition (MZT). Experimental evidence shows that NSUN5-dependent m^5^C stabilizes maternal mRNAs during folliculogenesis, and genetic ablation of NSUN5 results in follicular arrest, decreased ovarian function, and impaired embryonic development [78,79].

In parallel, YBX1 has been identified as a crucial m^5^C reader that binds to and stabilizes modified maternal transcripts, ensuring their availability before translational activation; loss of YBX1 interferes with maternal mRNA decay, alternative splicing, and zygotic genome activation (ZGA), leading to early embryonic arrest [80,81]. Proteomic and functional studies further show that YBX1 acts as a gatekeeper during early follicle development by repressing the translation of maternal mRNAs, with its downregulation enabling the coordinated activation of follicle growth [82]. At the level of translational control, maternal YBX1 also acts as a global translational repressor during oocyte maturation, and its dysregulation leads to excessive protein synthesis and defective embryogenesis [83]. Notably, recent evidence identifies a PGC7–AKT1–YBX1 signaling axis that alleviates YBX1-mediated repression through phosphorylation, thereby promoting maternal mRNA translation and supporting oocyte maturation and early embryonic development [84].

In addition to NSUN5, other m^5^C methyltransferases such as NSUN2 and NSUN6 contribute to maternal mRNA regulation across species, affecting cell cycle progression, MZT, and the conservation of early developmental programs [85]. Moreover, m^5^C-dependent RNA–protein interactions are essential for germline stem cell maintenance, as shown by the YBX1 homolog YPS, which selectively recognizes m^5^C-modified transcripts to regulate stem cell proliferation and differentiation [86]. A recent study found that NSUN2, NSUN5, NSUN6, and YBX1 are expressed in theca and granulosa cells in human ovarian tissue, along with many other genes. However, the mechanisms underlying their function in the ovary and their biological effects remain poorly understood [87].

Taken together, these findings highlight that m^5^C-mediated epitranscriptomic regulation, through the coordinated actions of writers, readers, and associated signaling pathways, ensures proper timing of maternal mRNA stability, translation, and clearance, thereby safeguarding oocyte quality, follicular development, and early embryonic competence (Table 1).

### 4.2. Physiological Roles of 5-methylcytosine (m^5^C) in Fertilization, Early Embryogenesis, and Endometrial Receptivity

Recent advances in in vitro fertilization techniques have enabled a more detailed examination of human embryos. After fertilization, the egg and sperm fuse to form a zygote, a single cell with two pronuclei. The zygote undergoes rapid mitotic divisions from the 2-cell to the 8-cell stage over three days, forming a cleavage-stage embryo. Once the embryo reaches approximately 16 to 32 cells, it forms a compact mass called the morula. The morula continues to divide, differentiate, and accumulate fluid, transforming into the blastocyst. The blastocyst consists of an outer layer of cells that develop into the trophoblast, which will become the placenta, and an inner cell mass that develops into the actual embryo. Fertilization and early embryonic development occur in the fallopian tubes. The blastocyst moves to the endometrial cavity and implants around day 6 or 7 after fertilization. This is followed by gastrulation with further embryonic differentiation and growth [88]. Early embryonic development involves many unique phenomena, including epigenetic programming and the maternal-to-zygote transition. The zygote depends entirely on maternal transcripts present in the egg at fertilization. During this period, the embryo’s DNA remains transcriptionally inactive, and maternal transcripts regulate the earliest stages of development, from initial cell divisions to the morula stage. The Maternal-to-Zygotic Transition (MZT) is characterized by maternal mRNA decay and ZGA. While the role of epigenetic reprogramming during gametogenesis (PGC) and preimplantation embryo development (blastocyst) is well documented, the role of epitranscriptomics throughout the mammalian life cycle remains understudied [89].

m^5^C plays an essential role during early embryogenesis, particularly in MZT, when control of gene expression shifts from maternal transcripts to the embryonic genome. Cross-species developmental mapping studies demonstrate that NSUN2 and NSUN6 regulate maternal mRNA and cell cycle progression [85]. The m^5^C reader YBX1 stabilizes modified maternal mRNAs by forming complexes with partners such as PABPC1a, protecting them from degradation and ensuring proper cleavage-stage development [80]. YBX1 also coordinates maternal mRNA decay, alternative splicing, and ZGA, with its loss leading to embryonic arrest at early stages [81]. Functionally, maternal YBX1 acts as a translational repressor to prevent premature protein synthesis, while signaling pathways such as PGC7–AKT1 modulate its activity to enable timely translation during development [84]. This m^5^C-dependent regulatory mechanism is evolutionarily conserved, as shown by the YBX1 homolog YPS in Drosophila, which controls germline stem cell maintenance and differentiation [86].

Beyond RNA stability and translation, m^5^C also regulates signaling pathways critical for embryonic development. NSUN5-mediated m^5^C modification has been shown to influence Hippo signaling by modulating YAP1 nuclear translocation and downstream gene expression, thereby regulating cell proliferation, apoptosis, and lineage specification in preimplantation embryos; NSUN5 depletion significantly reduces blastocyst formation and developmental competence [79]. In parallel, YBX1 has been implicated in regulating ZGA through interactions with m^6^A machinery, including METTL3 and IGF2BP1, linking m^5^C readers with other RNA modification pathways that coordinate maternal mRNA decay and embryonic genome activation [90]. Beyond development, m^5^C mediates fetal responses to environmental stress. For example, carbon black nanoparticle exposure induces fetal lung fibrosis via an NSUN2–m^5^C mechanism, where YBX1 stabilizes SP1 mRNA, activating DDRGK1 and downstream pathways that promote epithelial–mesenchymal transition (EMT) and abnormal lung development [91].

Differentiated cell nuclei can be reprogrammed through nuclear transfer (NT) into oocytes, resulting in cloned embryos. However, NT embryo development is frequently impeded by reprogramming failures, including incomplete activation of developmentally essential genes. Among H3K9me3-repressed, reprogramming-resistant loci, the m^5^C regulator ALYREF was identified as a critical factor. CRISPR/Cas9-mediated knockout of *Alyref* in mouse NT embryos caused early developmental arrest. Mechanistically, ALYREF is required for proper inner cell mass formation by regulating Nanog, and its supplementation via mRNA restores efficient preimplantation development. The partial silencing of *Alyref* in NT embryos is associated with H3K9me3-mediated repression of *Klf16* [92]. These findings highlight that failure to activate key m^5^C regulators can impair early embryonic development, emphasizing the essential role of coordinated epigenetic and epitranscriptomic control during preimplantation stages.

Endometrial receptivity denotes the window of implantation, during which the endometrial lining undergoes decidualization under the influence of progesterone in preparation for embryo implantation. Many genetic, epigenetic, and immune factors contribute to the establishment of a receptive endometrium that supports embryo adhesion, attachment, invasion, and placentation [93]. Emerging evidence indicates that m^5^C modification also plays a critical role in regulating endometrial function. In human endometrial tissues and in vitro models, NSUN2-mediated m^5^C methylation modulates gene expression, alternative splicing, and chromatin states through crosstalk with histone modifications. Specifically, NSUN2 overexpression promotes exon skipping of key genes such as *STAT1* and *MMP14* and enhances *CLDN4* expression via SIRT4/H3K9ac signaling, ultimately impairing embryo attachment and reducing endometrial receptivity [94].

Taken together, accumulating evidence across multiple model systems, including zebrafish, mice, and humans, supports a conserved role for m^5^C in regulating maternal mRNA stability, translational control, and developmental signaling pathways during fertilization, early embryogenesis, and implantation. Dysregulation of these processes, including the failure to activate H3K9me3-repressed, developmentally essential m^5^C regulator genes such as *Alyref*, can impair ZGA, disrupt embryo development, and compromise endometrial receptivity, ultimately contributing to reproductive failure (Table 1).

### 4.3. Polycystic Ovary Syndrome

Polycystic ovary syndrome (PCOS) provides direct mechanistic evidence linking m^5^C dysregulation to inflammatory ovarian pathology. PCOS is characterized by ovarian dysfunction, chronic low-grade inflammation, and metabolic disturbance. A recent study identified NSUN7 as a critical m^5^C writer in granulosa cells [95]. NSUN7 enhances m^5^C methylation of NLR family pyrin domain containing 3 (NLRP3) mRNA, increasing its stability and promoting inflammasome activation and pyroptotic cell death. Silencing NSUN7 reduces NLRP3 expression, attenuates granulosa cell pyroptosis, restores hormonal balance, and ameliorates disease progression in PCOS models. This work establishes the NSUN7–NLRP3 axis as a direct epitranscriptomic driver of inflammatory injury in PCOS (Table 1).

### 4.4. Premature Ovarian Insufficiency

Premature ovarian insufficiency (POI) is characterized by an accelerated depletion of primordial follicles, leading to the loss of normal ovarian function before age 40. Although the cause of POI remains unknown in many cases, some instances are associated with chromosomal abnormalities and genetic mutations [96]. More than 100 genes, including mutations on the X chromosome and autosomes, have been associated with POI [97].

Emerging evidence suggests that epitranscriptomic regulation, particularly m^5^C RNA methylation, plays a critical role in ovarian dysfunction linked to POI. In human granulosa cells, YBX1 has been identified as a key m^5^C reader that stabilizes m^5^C-modified transcripts of cell cycle–related genes; loss of YBX1 leads to destabilization of these transcripts, impaired G1–S transition, and causes granulosa cell cycle arrest, ultimately contributing to ovarian dysfunction and POI development [98]. Additionally, oxidative stress–induced ovarian damage is a significant mechanism underlying POI. DNMT2-mediated m^5^C modification has been shown to protect granulosa cells from reactive oxygen species (ROS)-induced DNA damage and apoptosis by facilitating DNA repair processes; disruption of this pathway compromises cell survival and accelerates ovarian failure [99].

Recent studies further highlight therapeutic potential through m^5^C-dependent pathways. In models of oxidative stress–induced ovarian dysfunction, exosome-derived YBX1 cooperates with DNMT2-mediated m^5^C modification to stabilize key mitochondrial transcripts such as Cytochrome C Oxidase Suunit 5B (*COX5B*), thereby reducing ROS accumulation, improving mitochondrial function, and alleviating granulosa cell senescence [100]. Collectively, these findings indicate that dysregulation of m^5^C-mediated RNA stability and stress response pathways contributes to granulosa cell dysfunction and follicular depletion, providing new mechanistic insights into the pathogenesis of POI (Table 1). These findings suggest that targeting m^5^C regulatory pathways may represent a novel therapeutic strategy for PCOS.

### 4.5. Pregnancy and Placental Biology: Preeclampsia as a Model Disorder

Pregnancy imposes profound transcriptional and post-transcriptional demands, particularly within the placenta, where trophoblast differentiation, vascular remodeling, and immune adaptation must be precisely coordinated. Emerging transcriptome-wide m^5^C profiling studies demonstrate altered m^5^C peak distribution in placental tissues from preeclampsia compared with normotensive pregnancies [101]. These changes are associated with dysregulated DNMT3B and TET3 expression and with perturbations in MAPK/cAMP signaling and hypoxia- and inflammation-related pathways, suggesting that m^5^C reprogramming contributes to placental dysfunction.

Further evidence from decidual tissues indicates that aberrant m^5^C methylation alters expression of genes involved in decidualization, disrupting key signaling pathways required for proper implantation and placental development [102]. Consistent with this concept, recent work has shown that NSUN2-mediated m^5^C methylation regulates genes involved in endometrial receptivity and decidualization, thereby influencing implantation success and early pregnancy establishment. These findings highlight an emerging role of RNA methylation in coordinating endometrial remodeling and maternal-embryo interactions during gestation [94].

Mechanistic studies strengthen the causal link between m^5^C and trophoblast function. Under hypoxic conditions, ALKBH1-mediated m^5^C demethylation destabilizes Proteasome 26S Subunit, Non-ATPase 14 (PSMD14) mRNA, thereby impairing trophoblast migration and invasion [103]. Conversely, enoxaparin treatment has been shown to activate the NSUN2/m^5^C/PAX3 axis, stabilizing PAX3 mRNA and restoring trophoblast function in vitro and in mouse models of preeclampsia, thereby improving hypertension, proteinuria, and placental injury [104]. These findings position m^5^C as a functional regulator of trophoblast invasion and placental homeostasis.

The placenta also functions as a central mediator of maternal–fetal communication. Placenta-derived microRNAs are packaged into extracellular vesicles (EVs) and transported into maternal circulation, where they regulate immune and metabolic adaptation [105]. RNA modifications influence RNA stability, export, and intercellular signaling capacity. Although direct evidence for m^5^C-modified extracellular vesicle RNAs remains limited, emerging perspectives propose that maternal environmental factors may shape fetal phenotypes through epitranscriptomic mechanisms, potentially including m^5^C [106] (Table 1).

**Table 1:** m^5^C RNA Modifications in Female Fertility, Benign Gynecology, and Pregnancy.

Reproduction and Development	Sample Types	Approaches	Mechanism	Biological Effects	Publication Date	Ref.
Embryonic development	Mammalian ovarian follicles and oocytes (mouse, pig, and cow)	Immunofluorescence and confocal microscopy to localize RNA modification machinery, assessment of m^6^A and m^5^C abundance in oocyte vs. somatic mRNA	Epitranscriptomic regulation of maternal mRNAs involves enzyme localization, with high m^6^A and lower m^5^C levels, controlling mRNA stability and translation during folliculogenesis and early embryonic transition.	Regulation of maternal mRNA stability and utilization, coordination of oocyte maturation and follicle development, and establishment of species-specific control of early embryonic development during transcriptional silencing	May, 2023	[73]
Ovarian dysfunction with reduced follicle count and ovarian aging	*Nsun5* KO mouse ovaries/oocytes and human granulosa-like tumor cell line (KGN) cells	m^5^C profiling, mRNA stability and splicing analysis, and functional validation	NSUN5-dependent m^5^C stabilizes maternal mRNAs and regulates translation during folliculogenesis and MZT	Follicular arrest, reduced ovarian function, and impaired embryonic development	Dec, 2022	[78]
Early embryonic development (mouse preimplantation embryos)	Mouse preimplantation embryos (two-cell to blastocyst stages)	siRNA-mediated knockdown of *Nsun5*; assessment of blastocyst formation, cell number, apoptosis, lineage markers (CDX2/OCT4), and YAP1 localization	NSUN5 regulates Hippo signaling by affecting YAP1 nuclear translocation and expression of LATS1/2, likely via m^5^C-dependent RNA regulation	*Nsun5* depletion reduces blastocyst formation, cell proliferation, and differentiation, increases apoptosis, and disrupts lineage specification	May, 2024	[79]
Early embryogenesis	Zebrafish early embryos	Genome-wide m^5^C mapping and YBX1-mediated RNA interaction analysis	m^5^C-modified maternal mRNAs are preferentially recognized by YBX1 through specific residue-mediated interactions, and together with Pabpc1a, YBX1 stabilizes these transcripts, thereby preventing their degradation during the MZT.	Maintains maternal mRNA stability and ensures successful early embryonic development during the transition from maternal to zygotic control.	Sep, 2019	[80]
Early embryonic development during pre-implantation	Mouse, human, bovine, and goat pre-implantation embryos; 8-cell stage embryos used for YBX1 knockdown studies	RNA-seq analysis of public datasets and YBX1 knockdown embryos (siRNA microinjection), differential gene expression analysis, alternative splicing (AS) analysis, enrichment analysis, 5-EU staining, and immunofluorescence	YBX1 regulates maternal mRNA stability and alternative splicing, mediates decay of maternal transcripts, and supports transcriptional activation during MZT	YBX1 ensures maternal mRNA decay, splicing, and ZGA; its knockdown arrests embryos at 2–4 cells with impaired decay and reduced blastocyst formation.	Feb, 2022	[81]
Zebrafish folliculogenesis	Zebrafish ovarian follicles at primary growth (PG) and secondary growth (SG) stages	Proteomic analysis of folliculogenesis (PG–SG transition), gene/protein expression profiling, phylogenetic analysis, and functional characterization of YBX1	YBX1 stabilizes and represses maternal mRNAs in early follicles; its downregulation releases these mRNAs for translation, enabling follicle activation.	Maintenance of early follicle development through mRNA stabilization and translational repression, followed by activation of follicle growth via coordinated mRNA translation	Oct, 2023	[82]
Embryonic development	Zebrafish oocytes and early embryos (maternal *ybx1* mutant and knockdown models)	Genetic knockout (null allele) and morpholino knockdown of YBX1, assessment of oocyte maturation and embryogenesis, translation activity assays, and rescue experiments via translational inhibition	Maternal YBX1 functions as a global translational repressor by associating with target mRNAs and processing body components, thereby limiting protein synthesis during oocyte maturation and early embryogenesis	YBX1 ensures oocyte maturation and MZT; its loss causes increased translation, stress response activation, and defective early embryonic development.	Oct, 2018	[83]
Oocyte maturation and early embryonic development	Mouse oocytes; maternal mRNA; genetic and molecular perturbation models	Functional studies including PGC7 depletion, protein interaction assays, phosphorylation analysis, translation assays, and AKT signaling pathway investigation	PGC7 promotes maternal mRNA translation by enabling AKT1 to phosphorylate YBX1, releasing translational repression on target mRNAs and forming the PGC7–AKT1–YBX1 axis.	Enhanced translation of maternal mRNAs, proper oocyte maturation, and support of early embryonic development through activation of key regulatory proteins	Dec, 2024	[84]
Embryonic development	Embryos across multiple species including Drosophila and humans	Developmental m^5^C mapping, evolutionary comparison, and functional genetic studies	NSUN2/NSUN6-mediated m^5^C regulates maternal mRNA dynamics during early embryogenesis	Controls cell cycle progression, maternal-to-zygotic transition, and evolutionary innovation of mRNA regulation	May, 2022	[85]
Ovarian germ line stem cell development	*Drosophila* ovarian germ line stem cells (GSCs); germ cells;	Loss- and gain-of-function of YPS, RNA-binding assays, m^5^C recognition analysis, cross-species rescue experiments with YBX1, and structural analysis of protein–RNA interactions	The YBX1 homolog YPS preferentially recognizes and binds m^5^C-modified RNAs via conserved cold-shock domains, enabling selective regulation of target mRNAs required for germ line stem cell maintenance and differentiation	Promotion of germ line stem cell maintenance, proliferation, and differentiation in the ovary; disruption of m^5^C binding impairs normal stem cell development	Feb, 2020	[86]
Early zygotic development	Porcine embryos, from the one-cell stage to blastocyst stage	Knockdown of *YBX1* using siRNA microinjection at the one-cell stage; additional knockdown of IGF2BP1 to study mechanistic links. Analysis included mRNA expression, histone modification, and blastocyst formation rate.	YBX1 regulates ZGA through m6A RNA modification by modulating the levels of the m^6^A writer METTL3 and m^6^A reader IGF2BP1, affecting maternal mRNA decay and ZGA gene expression	*YBX1* knockdown impairs blastocyst formation and ZGA by increasing maternal mRNA and disrupting m^6^A-mediated regulation	Jul, 2023	[90]
Fetal pulmonary fibrosis induced by maternal exposure to carbon black nanoparticles	In vivo pregnant rat exposure model with analysis of offspring lung tissues, as well as ex vivo lung epithelial cells	Animal exposure experiments, cell-based assays, gene expression and transcriptional regulation analyses, and functional studies to evaluate EMT, reticulophagy, and m^5^C RNA methylation	NSUN2-mediated m^5^C methylation enhances the stability of SP1 mRNA through recognition by YBX1, leading to increased SP1 protein expression; SP1 then transcriptionally activates DDRGK1, which interacts with FAM134B to promote reticulophagy, thereby driving EMT-associated fibrotic processes.	Increased reticulophagy, enhanced EMT, and aggravated fibrosis in offspring lung tissue following maternal nanoparticle exposure	Mar, 2025	[91]
Early development arrest	Mouse nuclear transfer embryos	H3K9me3 profiling to identify reprogramming-resistant genes, siRNA screening, CRISPR/Cas9 knockout of candidate genes, and mRNA supplementation experiments	ALYREF regulates Nanog to support proper inner cell mass formation, Alyref geneis partially silenced due to H3K9me3-mediated repression of *Klf16*	Knockout of *Alyref* results in early developmental arrest, whereas supplementation of its mRNA promotes efficient preimplantation development of cloned embryos	Aug, 2023	[92]
Recurrent Implantation. Failure, endometrial decidualization	Human endometrial tissues from proliferative and secretory stages, along with in vitro models including Ishikawa cells and BeWo spheroids to simulate embryo attachment.	Single-cell RNA-seq analysis, m^5^C-BS-seq, and dot blot assays to assess m^5^C levels, embryo attachment by Ishikawa–BeWo co-culture, with mechanisms further examined using Cut&Tag and co-immunoprecipitation.	NSUN2-mediated m^5^C regulates gene expression and splicing. Its overexpression increases STAT1 and MMP14 via exon skipping and enhances CLDN4 expression by modulating SIRT4 and H3K9ac, linking RNA methylation to histone modification.	*NSUN2* overexpression promotes proliferation and autophagy while reducing embryo attachment, impairing endometrial receptivity and contributing to recurrent implantation failure.	Apr, 2024	[94]
PCOS	PCOS patient samples, lipopolysaccharide (LPS)-treated KGN granulosa cell line; PCOS rat model ovaries and primary granulosa cells	LPS-induced pyroptosis model, ELISA, flow cytometry, Western blotting, MeRIP, dual-luciferase assay, actinomycin D treatment, and in vivo hormone analysis.	NSUN7 mediates m^5^C methylation of NLRP3 mRNA, enhancing its stability and promoting pyroptosis in granulosa cells.	Silencing *NSUN7* reduces granulosa cell pyroptosis, restores hormone balance, and attenuates PCOS progression; NLRP3 overexpression reverses these protective effects.	Oct, 2025	[95]
Primary ovarian insufficiency	Human granulosa cells (GCs) from POI patients, KGN human granulosa cell line	YBX1 knockdown and overexpression, Transcriptome and m^5^C methylome profiling	YBX1 stabilizes m^5^C-modified transcripts of cell cycle–related genes. Loss of YBX1 destabilizes these transcripts in an m^5^C-dependent manner, leading to impaired G1–S transition and cell cycle arrest.	Reduced granulosa cell proliferation, cell cycle arrest (G1 phase), granulosa cell dysfunction, contributes to POI pathogenesis	May, 2025	[98]
Premature ovarian failure.	Granulosa cells exposed to reactive oxygen species (ROS), particularly through H_2_O_2_-induced oxidative stress models	DNMT2 overexpression and mutational analysis, along with assays to evaluate ROS levels, DNA damage, apoptosis, and RNA m^5^C methylation activity	ROS induces nuclear translocation of DNMT2, which facilitates DNA damage repair in an m^5^C methylation–dependent manner, whereas impaired DNMT2 activity reduces RNA m^5^C modification and compromises DNA repair capacity	DNMT2 reduces ROS-induced DNA damage and apoptosis in granulosa cells, thereby protecting against cell death and contributing to the prevention or alleviation of premature ovarian failure.	Jun, 2021	[99]
Premature ovarian insufficiency (ROS-induced ovarian dysfunction and granulosa cell senescence)	Human granulosa cells (GCs) under oxidative stress, human umbilical cord mesenchymal stem cell-derived exosomes (HuMSCs-Ex)	Exosome-based therapeutic treatment combined with molecular, biochemical, and functional assays to investigate m^5^C RNA modification, including relevant analyses of DNMT2, YBX1, and COX5B	DNMT2 catalyzes m^5^C modification on *COX5B* mRNA, which is recognized by exosome-delivered YBX1 that directly binds and stabilizes the transcript, thereby promoting its translation in an m^5^C-dependent manner	Reduced ROS accumulation, improved mitochondrial function, decreased granulosa cell senescence, and overall alleviation of oxidative damage associated with POI	May, 2025	[100]
Preeclampsia	placental tissues from normotensive pregnancies and preeclampsia patients	UMI-MeRIP-Seq transcriptomic m^5^C profiling and pathway enrichment analysis	Dysregulated m^5^C distribution and altered DNMT3B/TET3 expression affect MAPK/cAMP and hypoxia/inflammatory pathways	Contributes to placental dysfunction and PE-associated immune and hypoxic responses	Feb, 2023	[101]
Preeclampsia	Human decidual tissue from PE and normal pregnancy	MeRIP-seq and RNA-seq with integrative bioinformatics, pathway analysis, and qPCR validation	Aberrant m^5^C methylation alters mRNA expression of decidualization-related genes, disrupting key signaling pathways in PE	Distinct methylation and gene expression signatures, identification of PE-associated genes, and impaired decidualization collectively contribute to disease development.	Nov, 2023	[102]
Preeclampsia	Hypoxia-treated trophoblast cells	ALKBH1 modulation with functional assays and m^5^C/RNA interaction analyses	ALKBH1-mediated m^5^C demethylation destabilizes PSMD14 mRNA, impairing trophoblast function ALKBH1-mediated m^5^C demethylation destabilizes PSMD14 mRNA, impairing trophoblast function	Reduced trophoblast viability, migration, and invasion contributing to PE pathogenesis	Mar, 2025	[103]
Preeclampsia	Hypoxic trophoblast cells, PE placentas, and PE mice	Enoxaparin treatment with m^5^C analysis, NSUN2/PAX3 interaction assays, and functional validation in vitro and in vivo	Enoxaparin activates the NSUN2/m^5^C/PAX3 axis to stabilize PAX3 mRNA and restore trophoblast function	Improved trophoblast activity and amelioration of PE-associated hypertension, proteinuria, and placental injury	Nov, 2025	[104]

Across physiological development and reproductive disease, m^5^C functions as a regulator of RNA stability, translational efficiency, inflammatory signaling, trophoblast invasion, placental vascular integrity, and hormonal responsiveness. Mechanistic evidence is strongest in early embryogenesis, preeclampsia, and PCOS, while emerging data suggests broader roles in endometriosis and fibroid biology. Collectively, current findings position m^5^C as a critical layer of post-transcriptional control in women’s reproductive health, integrating developmental timing, immune regulation, endocrine signaling, and disease susceptibility.

## 5. m^5^C in Women-Specific Cancers

Emerging evidence implicates dysregulation of m^5^C RNA modification in the initiation, progression, and therapeutic resistance of multiple cancers that disproportionately affect women. Alterations in the expression or activity of m^5^C writers and readers can reshape post-transcriptional gene regulation, promoting oncogenic phenotypes such as uncontrolled proliferation, invasion, and immune evasion. Given the strong influence of hormonal signaling in many female cancers, m^5^C-mediated RNA regulation represents a critical intersection between epitranscriptomics and hormone-dependent tumor biology.

### 5.1. Breast Cancer

m^5^C RNA methylation plays a central and multifaceted role in breast cancer (BC), integrating transcriptional regulation, subtype specification, metabolic reprogramming, tumor progression, and therapeutic resistance. Among m^5^C writers, the RNA methyltransferase NSUN2 is the most extensively characterized in BC. NSUN2 is frequently overexpressed in breast tumors and correlates with increased tumor growth, metastasis, and poor patient prognosis. Mechanistically, NSUN2-mediated m^5^C deposition enhances the stability and translational efficiency of mRNAs encoding oncogenic and cell-cycle–related proteins, thereby promoting proliferative and survival advantages. In addition, NSUN2 contributes to stress adaptation and therapy resistance, thereby supporting tumor plasticity in hostile microenvironments [17,25,107,108].

At the molecular level, the NSUN2/YBX1 axis represents a core regulatory mechanism. NSUN2 functions as an m^5^C “writer,” while YBX1 acts as a key “reader,” recognizing m^5^C-modified transcripts and enhancing their stability and translation. Transcriptome-wide analyses reveal subtype-specific m^5^C methylation landscapes that reshape gene expression networks and increase global protein synthesis [107,109]. Expanding this framework, NSUN6 has recently been shown to catalyze high-stoichiometry, site-specific m^5^C modification on a subset of mRNAs harboring CUCCA motifs within stem–loop structures. These modified transcripts are preferentially recognized by m^5^C readers YBX1 and YBX3, resulting in enhanced mRNA stability and increased BC cell migration, highlighting a cooperative NSUN6–YBX1/YBX3 regulatory axis with potential implications for therapeutic mRNA stabilization [110]. In triple-negative BC (TNBC), NSUN2 also modifies tRNAs, promoting codon-biased translation of glycolysis-related genes, driving metabolic rewiring, invasion, and docetaxel resistance [108] (Table 2).

BC subtypes differ markedly in their dependence on estrogen and progesterone signaling, and m^5^C regulation appears to vary accordingly [146]. In hormone receptor–positive tumors, estrogen signaling influences RNA metabolism and may indirectly modulate expression or activity of m^5^C machinery. Early mechanistic studies further demonstrated that NFIB and YBX1 interact with the ESR1–FOXA1 transcriptional complex, suppressing ESR1 transactivation and promoting an estrogen-independent phenotype [111], thereby contributing to endocrine escape and progression toward ER-negative states. In contrast, hormone receptor–negative and TNBC subtypes exhibit heightened reliance on post-transcriptional regulation, including RNA modification pathways, to sustain aggressive behavior, suggesting that m^5^C may represent a subtype-specific vulnerability.

Beyond primary tumor progression, m^5^C signaling plays a pivotal role in metastatic dissemination. In BC brain metastasis (BCBM), NSUN2 mediates m^5^C methylation of circRNAs such as hsa_circ_0004516, enhancing their stability in a catalytic-dependent manner and activating AKT signaling, thereby establishing the NSUN2–m^5^C–circRNA–AKT axis that promotes proliferation, migration, invasion, and brain metastatic progression [112]. In parallel, NSUN2-mediated m^5^C modification of PSD4 mRNA enhances its stability and nuclear export via YBX1 binding, leading to ferroptosis resistance, vasculogenic mimicry, and metastatic outgrowth through the NSUN2–m^5^C–PSD4–ferroptosis axis [113]. Similarly, in BC bone metastasis, NSUN2 regulates tumor–bone microenvironment interactions through epitranscriptomic control. NSUN2-mediated m^5^C modification of KDM6B mRNA promotes its PPIA-dependent degradation, resulting in downstream NUMB hypermethylation, activation of Notch signaling, and upregulation of RANKL. This NSUN2–KDM6B–Notch axis enhances osteoclast differentiation and drives osteolytic bone metastasis, underscoring the role of m^5^C in metastatic niche remodeling [114].

Beyond transcriptional adaptation, m^5^C-dependent signaling intersects with major oncogenic pathways regulating and autophagyolism and autophagy. In TNBC, SAT1 stabilizes YBX1 via deubiquitination, reinforcing m^5^C-mediated stabilization of mTOR mRNA and suppressing autophagy, thereby promoting tumor aggressiveness [115]. Moreover, elevated m^5^C levels contribute to endocrine resistance, as NSUN7-mediated reprogramming enhances tamoxifen resistance, migration, clonogenicity, and poor prognosis [116]. Notably, NSUN6 demonstrates context-dependent functions, acting as a potential tumor suppressor in ovarian cancer (OC) but promoting migration in BC, highlighting tissue-specific m^5^C regulatory dynamics [117].

Collectively, m^5^C reprogramming in BC drives estrogen-dependent and estrogen-independent, subtype-specific transcriptional adaptation, metabolic remodeling, enhanced proliferation and metastasis, autophagy suppression, and resistance to chemotherapy and endocrine therapy. Its additional roles in regulating circRNA stability, ferroptosis resistance, and tumor–microenvironment interactions further expand its importance in metastatic progression. These findings position m^5^C regulatory pathways as promising targets for precision therapeutic strategies across BC subtypes (Table 2).

### 5.2. m^5^C in Gynecologic Malignancies

#### 5.2.1. Ovarian Cancer

OC is characterized by marked molecular heterogeneity and extensive dysregulation of RNA processing pathways, including m^5^C-mediated epitranscriptomic remodeling. Elevated expression of m^5^C writers, particularly NSUN2, has been consistently associated with enhanced proliferation, invasion, metastasis, and chemoresistance in OC models. Large-scale TCGA-based analyses further demonstrate that expression of m^5^C regulators defines distinct molecular subtypes with differential survival outcomes and immune microenvironments [118,119,120,121]. Aberrant m^5^C patterns reshape key oncogenic pathways, including Wnt/β-catenin, PI3K–Akt, focal adhesion signaling, and metabolic networks, thereby contributing to tumor progression and interpatient heterogeneity.

**Figure 2:**
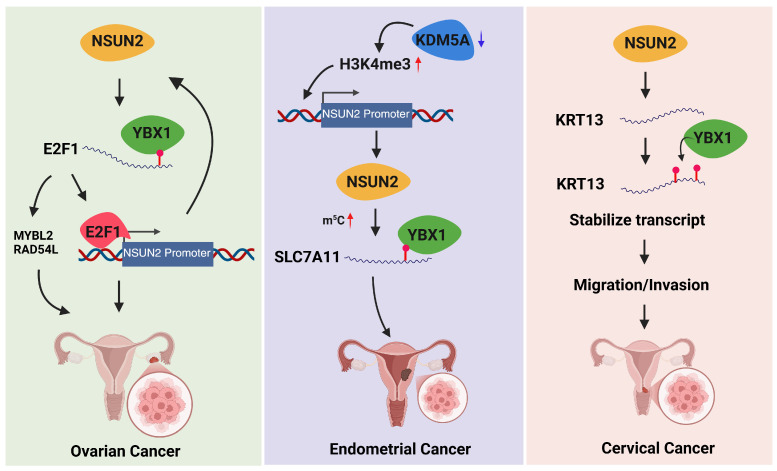
**Proposed mechanisms of NSUN2 in gynecological cancers.** Schematic illustration summarizing the regulatory roles of NSUN2 across cervical, ovarian, and endometrial cancers. **Left panel (ovarian cancer):** NSUN2 forms a regulatory loop with YBX1 and E2F1. NSUN2-mediated RNA methylation stabilizes YBX1-associated transcripts, facilitating E2F1 activation. E2F1 in turn promotes transcription of cell cycle–related genes (e.g., MYBL2 and RAD54L) and enhances NSUN2 promoter activity, thereby establishing a positive feedback circuit that drives ovarian cancer proliferation [122]. **Middle panel (endometrial cancer):** Epigenetic regulation by KDM5A modulates H3K4me3 levels at the NSUN2 promoter, increasing NSUN2 transcription. Elevated NSUN2 catalyzes m^5^C methylation of SLC7A11 mRNA, which is recognized by YBX1, enhancing transcript stability and expression [135]. This pathway contributes to metabolic adaptation and tumor growth in endometrial cancer. **Right panel (cervical cancer):** NSUN2 enhances the translation of KRT13 mRNA through interaction with the RNA-binding protein YBX1 and m^5^C RNA methylation, leading to increased migration and invasion, and promoting cervical tumor progression [137]. Arrows indicate activation or promotion, and red dots represent m^5^C RNA methylation marks. The uterus icons depict the associated tumor type for each pathway.

An identified oncogenic mechanism in OC is the NSUN2–YBX1 positive feedback loop. NSUN2 mediates m^5^C methylation of *E2F1* mRNA, which is recognized by the m^5^C reader YBX1. This interaction promotes YBX1 phase separation and enhances E2F1 translation. In turn, E2F1 transcriptionally activates NSUN2, forming a self-amplifying loop that drives oncogenic transcriptional programs, including activation of MYBL2 and RAD54L, thereby accelerating tumor progression [122] (Figure 2).

Recent studies further expand the m^5^C regulatory landscape in OC. Multi-omics analyses of TCGA datasets identified epigenetic (m^5^C/m^6^A/m^1^A/m^7^G)-related immune lncRNA signatures that stratify prognostic subtypes and predict immune checkpoint and chemotherapy responses [123]. Comprehensive m^5^C profiling of lncRNAs in high-grade serous ovarian cancer (HGSOC) tissues demonstrated widespread dysregulation of m^5^C peaks, which altered PI3K–AKT and other cancer-related signaling pathways and were associated with increased tumor growth, invasion, and metastasis, as well as poor prognosis [124]. In HGSOC, NSUN1 overexpression increases global m^5^C methylation and enhances RAPGEF4 expression, thereby activating pro-tumorigenic signaling and promoting proliferation, migration, and invasion, supporting its potential as a diagnostic biomarker [125]. In anesthetic-associated tumor progression, sevoflurane exposure promotes DNMT2-mediated m^5^C modification of APC mRNA, activating β-catenin signaling and enhancing malignant behaviors, including proliferation, invasion, and tumor growth [126]. Moreover, ALYREF stabilizes m^5^C-modified BIRC5 mRNA to drive oncogenic processes, promoting OC proliferation, migration, invasion, glycolysis, and tumor growth, whereas its knockdown suppresses malignancy [127]. In addition to stabilizing oncogenic transcripts, ALYREF functions as a canonical m^5^C reader that facilitates nuclear export of methylated RNAs, thereby coupling RNA cytosine methylation to post-transcriptional RNA trafficking and translational efficiency in OC cells.

m^5^C modification also plays a pivotal role in platinum resistance in OC. Multiple mechanisms converge on the m^5^C–YBX1 axis: YBX1 binds m^5^C-modified CHD3 to enhance homologous recombination repair [128]; ALYREF stabilizes m^5^C-modified LGR4, activating Wnt/β-catenin signaling [129]; and YBX1/MATR3 cooperate to stabilize CCNL2, promoting cisplatin resistance [130]. Conversely, SIAH1-mediated ubiquitination of YBX1 promotes its degradation and reverses chemoresistance [131], underscoring YBX1 as a central determinant of therapeutic response in OC.

In addition to proliferation and drug resistance, m^5^C remodeling shapes the tumor immune microenvironment. m^5^C-related lncRNA signatures stratify patients by prognosis and immune infiltration patterns [132], while NSUN2-mediated stabilization of SNHG15 enhances PD-L1 expression and promotes immune evasion [133].

Collectively, m^5^C reprogramming in OC promotes oncogenic transcriptional amplification, DNA repair capacity and platinum resistance, immune escape, metabolic adaptation, EMT, ferroptosis suppression, and β-catenin pathway activation. These findings position the NSUN2–YBX1 axis and broader m^5^C regulatory networks as promising therapeutic and prognostic targets in ovarian malignancies (Table 2).

#### 5.2.2. Endometrial Cancer

Endometrial cancer (EC) arises in a hormonally responsive tissue that undergoes cyclical remodeling, rendering it particularly sensitive to post-transcriptional regulatory mechanisms. Emerging evidence indicates that m^5^C epitranscriptomic reprogramming plays a significant role in endometrial tumorigenesis by influencing RNA stability, translational control, ferroptosis regulation, and immune modulation. Aberrant expression of m^5^C machinery has been linked to dysregulated cell-cycle progression and inflammatory signaling pathways, suggesting that m^5^C modification may act as a molecular integrator of hormonal exposure, metabolic dysfunction, and oncogenic signaling in this estrogen-responsive malignancy [135].

A key mechanism in EC involves the NSUN2–YBX1–SLC7A11 axis. NSUN2-mediated m^5^C methylation stabilizes SLC7A11 transcripts through YBX1 recognition, suppressing ferroptosis and enhancing tumor cell survival and proliferation [135] (Figure 2). By promoting resistance to oxidative stress–induced cell death, this pathway supports tumor growth and therapeutic resistance, highlighting regulation of ferroptosis as a key functional consequence of m^5^C remodeling in EC.

Beyond intrinsic tumor growth control, m^5^C-related lncRNA signatures have demonstrated significant prognostic and immunologic relevance. Integrative transcriptomic analyses reveal that m^5^C-associated lncRNA expression profiles can stratify patients according to overall survival and are positively correlated with tumor grade, PD-L1 expression, and distinct immune cell infiltration patterns [134,136]. These observations indicate that m^5^C modification not only drives tumor cell proliferation and ferroptosis resistance but also contributes to remodeling of the tumor immune microenvironment, potentially influencing immune evasion and responsiveness to immunotherapy. Altered m^5^C landscapes may therefore shape cytokine signaling, checkpoint regulation, and interactions between tumor cells and infiltrating lymphocytes or macrophages in EC.

Collectively, m^5^C dysregulation in EC promotes sustained proliferation, resistance to ferroptotic cell death, metabolic resilience, and immune modulation. These converging effects position m^5^C regulatory networks as promising biomarkers for prognostic stratification and as potential therapeutic targets in this hormonally and metabolically influenced malignancy (Table 2).

#### 5.2.3. Cervical Cancer

Cervical cancer (CC), primarily cervical squamous cell carcinoma and cervical adenocarcinoma arising from the transformation zone of the uterine cervix, is closely associated with persistent high-risk human papillomavirus (HPV) infection [147]. This gynecologic malignancy exhibits highly active m^5^C-dependent epitranscriptomic reprogramming that influences tumor progression, therapy resistance, regulation of ferroptosis, and immune modulation. Given the viral etiology of this epithelial cancer, m^5^C dysregulation may affect both host and viral RNA metabolism, potentially regulating viral transcript stability and translation as well as host antiviral immune responses. Altered m^5^C patterns may therefore contribute to viral persistence, immune evasion, and malignant transformation of cervical epithelial cells [137] (Figure 2).

An identified oncogenic mechanism in cervical carcinoma involves the NSUN2–YBX1 axis, in which NSUN2-mediated m^5^C methylation stabilizes multiple oncogenic transcripts through YBX1 recognition. Key targets include LRRC8A [138], LINC01094 [139], and KRT13 [137], whose stabilization enhances tumor cell proliferation, EMT, metastatic potential, and resistance to apoptosis. These findings position NSUN2-driven m^5^C deposition as a major driver of aggressive phenotypes in HPV-associated cervical tumors.

m^5^C modification also contributes substantially to therapeutic resistance in CC. NSUN6-mediated m^5^C stabilization of NDRG1 via the reader protein ALYREF enhances homologous recombination–mediated DNA repair, promoting radioresistance in patients receiving radiotherapy [140]. Under hypoxic tumor microenvironment conditions, ALYREF further stabilizes KIF20A in an m^5^C-dependent manner, activating BUB1 and inducing ferroptosis resistance [141]. Additionally, m^5^C stabilization of SERPINB5 promotes activation of mitotic regulator and chemoresistance to agents such as paclitaxel and vincristine, effects that can be reversed upon target depletion [142].

Beyond intrinsic tumor growth and resistance mechanisms, m^5^C regulator–based clustering analyses in CC cohorts define molecular subtypes with distinct prognostic outcomes, immune infiltration patterns, and predicted responses to immunotherapy [143,144,145]. These data indicate that m^5^C remodeling in this HPV-driven gynecologic carcinoma not only drives EMT, metastasis, and radio- and chemoresistance, but also reshapes the tumor immune microenvironment.

Collectively, m^5^C reprogramming in CC integrates viral–host RNA regulation with oncogenic signaling, ferroptosis suppression, DNA repair enhancement, and immune modulation, highlighting m^5^C regulatory networks as promising therapeutic and prognostic targets in HPV-associated cervical malignancies (Table 2).

## 6. m^5^C RNA Modification in Metabolic and Cardiovascular Health in Women

**Figure 3:**
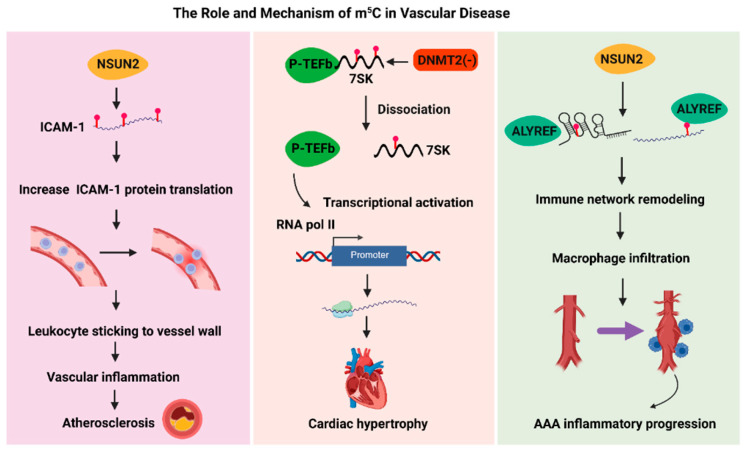
**Epitranscriptomic regulation of cardiovascular pathology by m^5^C RNA methylation.** Left panel: In vascular endothelial cells, the RNA methyltransferase NSUN2 deposits m^5^C modifications on ICAM-1 mRNA, enhancing its translational efficiency. Elevated ICAM-1 expression strengthens leukocyte adhesion to the endothelial surface, initiating a pro-inflammatory vascular niche that promotes sustained immune cell recruitment. This inflammatory amplification contributes to endothelial dysfunction and accelerates atherosclerotic lesion development [148]. Middle panel: In the myocardium, DNMT2 acts as a regulatory brake on transcriptional activation through stabilization of the inhibitory 7SK–P-TEFb complex. Loss of DNMT2 decreases 7SK m^5^C, and promotes dissociation of 7SK, resulting in hyperactivation of P-TEFb–dependent transcriptional programs that drive cardiomyocyte growth. This epitranscriptomic deregulation leads to maladaptive cardiac hypertrophy in the mouse model [149]. Right panel: In abdominal aortic aneurysm (AAA), coordinated upregulation of m^5^C regulators (NSUN2, NSUN5) and the reader protein ALYREF reshapes immune-associated RNA regulatory networks. Aberrant m^5^C signaling enhances macrophage infiltration and inflammatory remodeling of the aortic wall, establishing a self-reinforcing inflammatory microenvironment that accelerates aneurysm progression [150].

Metabolic homeostasis, including lipid, glucose, and energy balance, varies between sexes due to interactions between hormones (e.g., estrogen) and regulatory gene networks. While most work to date has focused on RNA modifications such as N^6^-methyladenosine (m^6^A) in metabolic diseases, transcriptome-wide studies and reviews highlight that m^5^C and other RNA methylations influence the expression of genes involved in lipid and glucose metabolism by altering RNA stability and translation, supporting possible roles for m^5^C in systemic metabolic regulation. For example, studies of metabolic dysfunction-associated steatotic liver disease report that m^5^C RNA methylation is enriched in lipid metabolism-related genes, linking m^5^C landscapes to pathways central to obesity and insulin resistance [151]. Although direct studies of m^5^C in women’s metabolic regulation remain limited, the broader epitranscriptomic literature supports a role for RNA modifications in systemic metabolic control [152].

### 6.1. Pregnancy-Associated Metabolic Disorders

Pregnancy introduces dramatic metabolic shifts to support fetal growth, including changes in carbohydrate and lipid metabolism. Dysregulation of these adaptations can lead to pregnancy-associated disorders such as gestational diabetes mellitus (GDM) and preeclampsia. Epitranscriptomic modifications have been identified in placental tissue from preeclampsia, where altered m^5^C peak distribution was associated with genes in signaling pathways linked to inflammation, hypoxia response, and metabolism; aberrations in m^5^C regulators were also observed in preeclampsia placentas [101]. This suggests that m^5^C dynamics may influence placental metabolic programming and maternal–fetal nutrient handling, processes central to metabolic adaptation during pregnancy. Altered placental RNA methylation could contribute to the pathogenesis of GDM and related outcomes, although direct functional data on m^5^C in gestational metabolic disorders are still emerging.

### 6.2. Cardiovascular Disease Risk Modulation

Cardiovascular disease (CVD), including atherosclerosis and cardiac remodeling, is a leading cause of mortality among women, accounting for nearly one-third of female deaths. Traditional risk factors such as diabetes, hypertension, obesity, and smoking have a disproportionately greater impact in women, alongside female-specific factors (e.g., pregnancy complications, menopause) and female-predominant conditions like autoimmune diseases [153]. Epitranscriptomic regulation, particularly m^5^C RNA methylation, is increasingly linked to CVD. Dysregulated m^5^C regulators affect cardiomyocyte function, vascular integrity, and mitochondrial processes, while m^5^C-mediated control of endothelial activation, inflammation, and smooth muscle cell plasticity contributes to atherosclerosis and vascular remodeling [154,155,156].

#### 6.2.1. m^5^C Reprogramming and Cardiovascular Diseases

m^5^C RNA modification undergoes dynamic reprogramming in various cardiovascular and vascular diseases. In cardiac regeneration, remodeling of m^5^C during pluripotency exit and cardiac lineage commitment regulates lineage-specific transcripts, supporting cardiomyocyte differentiation and maturation [157].

In vascular pathology, oxidative stress–induced nucleolar remodeling represents an additional m^5^C-linked mechanism. In human aortic vascular smooth muscle cells (VSMCs), curcumin downregulates SIRT7 while upregulating DNMT2, suppressing rDNA transcription and enhancing RNA methylation. This induces nucleolar stress, activates p53/p21 signaling, and promotes oxidation-dependent cytostasis, thereby limiting VSMC proliferation and potentially reducing atherosclerosis progression or post-angioplasty restenosis [158]. RNA cystosine methylation has been implicated in regulating inflammatory cytokine production, cholesterol metabolism, and macrophage polarization, processes that drive foam-cell formation and plaque progression [159,160]. Dysregulated m^5^C signaling in vascular immune cells may therefore contribute to chronic vascular inflammation and plaque instability.

In AAA, dysregulated m^5^C enhances lncRNA–mRNA networks, promoting immune cell infiltration and inflammatory signaling [150], while in pressure-overload remodeling, NSUN2-mediated m^5^C of NF-κB mRNA attenuates hypertrophy and fibrosis, improving cardiac function [161]. In ischemic and stress-related injury, m^5^C-dependent pathways regulate cardiomyocyte fate: piRNA–DNMT1 signaling alters ATF7 methylation to promote necroptosis in ischemia/reperfusion injury [162], whereas NSUN2-mediated m^5^C modification of NCOA4 suppresses ferroptosis sepsis-induced myocardial injury [163]. Additional studies show that disruption of ALYREF contributes to doxorubicin cardiotoxicity [164] and NSUN2-stabilization of HuR mRNA promotes post-myocardial infarction fibrosis [165], collectively highlighting m^5^C as a central regulator of cardiac injury and repair.

#### 6.2.2. m^5^C Regulators and Cardiovascular Diseases

NSUN2 plays a critical role in multiple cardiovascular conditions by regulating RNA stability and translation through m^5^C methylation. In vascular inflammation and allograft arteriosclerosis, NSUN2 methylates intercellular adhesion molecule 1 (ICAM-1) mRNA, enhancing its translation and promoting leukocyte adhesion, while NSUN2 deficiency reduces inflammation [148]. In doxorubicin-induced cardiotoxicity, NSUN2 methylates Nrf2 mRNA, increasing its stability and protein expression, thereby enhancing antioxidant stress and promotes cardiomyocyte survival [166]. During cardiac hypertrophy and heart failure, NSUN2 methylates PRKACA mRNA in a YBX1-dependent manner, sustaining PKA signaling and contractility; loss of NSUN2 impairs cardiac adaptation [167]. In hypoxic pulmonary hypertension, NSUN2-mediated m^5^C stabilizes circCCNL2, allowing it to sequester FXR2 and limit pulmonary artery smooth muscle cell proliferation [168].

NSUN3 contributes to atherosclerosis by enhancing macrophage inflammatory cytokine production, driving disease progression and serving as a potential diagnostic biomarker [169]. NSUN4 functions in mitochondrial cardiomyopathy by methylating 12S rRNA and, in complex with MTERF4, coordinating the assembly of mitochondrial ribosomal subunits; loss of NSUN4 abolishes mitochondrial translation and disrupts cellular energy homeostasis [14]. DNMT2 facilitates cardiac hypertrophy by methylating tRNAs and stabilizing RNAs, thereby promoting P-TEFb activation via 7SK RNA release and driving hypertrophic growth [149].

ALYREF serves as a key m^5^C reader in post-MI cardiac remodeling, where it binds target transcripts to enhance extracellular matrix protein synthesis, promoting fibroblast proliferation, collagen and elastin deposition, and overall cardiac remodeling [170]. In doxorubicin cardiotoxicity, disruption of ALYREF phase separation destabilizes the m^5^C reader complex, leading to increased DNA damage and apoptosis, while preservation of ALYREF mitigates cardiotoxicity [164]. YBX1 mediates m^5^C-dependent translational enhancement of PRKACA in cardiac hypertrophy and heart failure, sustaining PKA signaling and supporting cardiac adaptation [167].

TET3, as an m^5^C eraser, contributes to the pathogenesis of acute coronary syndrome by removing m^5^C modifications from the lncRNA CCAT2, which decreases its stability and releases TRIM14 mRNA from repression. This process promotes endothelial apoptosis and impairs proliferation and angiogenesis, thereby driving disease progression [171].

Collectively, m^5^C RNA methylation critically regulates cardiovascular disease by modulating RNA stability, translation, nucleolar integrity, mitochondrial function, inflammation, hypertrophy, fibrosis, and programmed cell death. Through coordinated actions of writers (NSUN2, NSUN3, NSUN4, DNMT2), readers (ALYREF, YBX1), and erasers (TET3), m^5^C reprogramming influences both vascular and myocardial pathophysiology. Targeting these m^5^C regulators offers promising opportunities for mechanistic insight, risk stratification, and therapeutic intervention in diverse cardiovascular disorders (Figure 3, Table 3).

#### 6.2.3. Sex Differences in Cardiovascular Disease and Clinical Implications of m^5^C Regulation

CVD exhibits well-established sex differences in disease onset, progression, and outcomes [171,172]. Women present distinct cardiovascular risk profiles shaped by hormonal status, pregnancy, menopause, and metabolic transitions. Traditional risk factors, including atherosclerosis, hypertension, diabetes, dyslipidemia, non-alcoholic fatty liver disease, and obesity, often exert greater impact during midlife and postmenopause [173]. In addition, CVD is closely linked to female-specific conditions such as UFs [174], preeclampsia [175], adverse pregnancy outcomes [176], PCOS [177,178], endometriosis [179], and premature menopause [180], underscoring the importance of sex-specific cardiovascular risk assessment.

Emerging evidence indicates that epitranscriptomic mechanisms, particularly RNA m^5^C modification, regulate pathways central to cardiac development, mitochondrial function, endothelial inflammation, fibrosis, and immune activation. These biological processes are strongly influenced by sex hormones and female-specific physiological transitions, suggesting that m^5^C reprogramming may contribute to sex-dependent cardiovascular phenotypes. Although direct evidence linking m^5^C alterations specifically to women’s vascular diseases in a sex-differentiated context remains limited, current knowledge of sex differences in vascular biology and immune regulation supports a plausible mechanistic connection. Future research should prioritize female-specific experimental models and hormone-aware study designs to clarify the role of m^5^C modification in women’s cardiovascular health and to advance precision medicine approaches tailored to female patients.

## 7. Clinical Implications and Translational Potential

Understanding and measuring m^5^C RNA modifications is not only mechanistically interesting, but growing evidence points to its utility as a biomarker for disease detection and prognosis, as well as its potential integration into liquid biopsy platforms leveraging circulating RNA.

### 7.1. m^5^C as Biomarkers

#### 7.1.1. Diagnostic and Prognostic Applications

m^5^C RNA modification patterns and their regulatory machinery have been associated with clinical outcomes in multiple disease contexts, especially cancer, but also in benign conditions.

The diagnostic performance of NSUN7 has been evaluated in patients with PCOS. Receiver operating characteristic (ROC) curve analysis of NSUN7 protein levels in granulosa cells demonstrated that NSUN7 may serve as a highly accurate diagnostic biomarker for PCOS [95]. Similarly, transcriptomic profiling and integrative models incorporating the expression of m^5^C regulators (e.g., writers and readers) have successfully stratified patient prognosis and correlated with disease progression in several tumor types. For example, m^5^C-based gene signatures have been developed to predict survival outcomes in CC and other malignancies, supporting the potential of m^5^C-related patterns as prognostic biomarkers [143].

More broadly, accumulating evidence indicates that dysregulated expression of m^5^C regulatory genes (e.g., NSUN family members and DNMT2) and disrupted m^5^C methylation profiles correlate with tumor stage, therapeutic response, and overall patient survival across multiple cancer types. These findings support the potential utility of measuring m^5^C regulators or global patterns of m^5^C modification as diagnostic and prognostic biomarkers in precision oncology [58].

#### 7.1.2. Circulating RNA and Liquid Biopsy Potential

Circulating nucleic acids, including cell-free RNA (cfRNA)**,** are increasingly being leveraged in non-invasive liquid biopsy approaches for disease detection and monitoring [181]. cfRNA encompasses RNA molecules shed into biofluids (e.g., blood) from tissues throughout the body, providing a rich source of disease-associated signals without the need for tissue biopsy.

Proof-of-principle studies indicate that levels of m^5^C modification in peripheral blood immune cells can discriminate disease states. In patients with colorectal cancer, m^5^C levels in blood RNA were significantly elevated compared with those in healthy controls, and m^5^C measurement achieved high diagnostic performance, outperforming traditional serum biomarkers such as carcinoembryonic antigen (CEA), CA19-9, and CA125 [182].

These findings demonstrate that m^5^C status in circulating RNA, whether in cfRNA from blood cells or potentially in EVs, holds strong promise as a non-invasive biomarker for early detection, disease monitoring, and prognostic evaluation. Advances in sequencing and modification detection technologies are beginning to enable more sensitive and specific measurement of RNA methylation signatures in liquid biopsies, pointing toward future clinical applications.

### 7.2. Therapeutic Targeting of m^5^C Machinery

Given the growing evidence that dysregulated m^5^C RNA modification contributes to cancer, metabolic disease, and inflammatory disorders, components of the m^5^C machinery are increasingly being explored as potential therapeutic targets. Strategies aimed at modulating m^5^C dynamics include small-molecule inhibitors of m^5^C writers or readers, RNA-based approaches to alter target transcripts, and indirect targeting through upstream regulatory pathways.

#### 7.2.1. Small-Molecule Inhibitors

NSUN family proteins (e.g., NSUN2, NSUN7) and DNMT2 are attractive therapeutic targets due to their enzymatic activity and disease-associated overexpression in cancer and inflammatory conditions. Small-molecule inhibitors could suppress aberrant m^5^C deposition, thereby reducing the stability or translation of pathogenic transcripts, including oncogenes or inflammatory mediators [183,184]. For example, NSUN2 drives multidrug resistance in anaplastic thyroid carcinoma by methylating SRSF6 mRNA, which is recognized by the reader ALYREF, thereby reprograming UAP1 splicing toward AGX2, stabilizing ABC transporters and promoting drug efflux. Pharmacologic inhibition of NSUN2 disrupts this pathway and enhances chemotherapy efficacy, highlighting m^5^C writers as promising targets in ATC [185]. Similarly, m^5^C readers such as YBX1 are critical for drug resistance in OC, where YBX1 recognizes m^5^C-modified CHD3 mRNA and stabilizes it via PABPC1, enhancing chromatin accessibility and homologous recombination–mediated DNA repair. Inhibition of YBX1 (e.g., SU056) reverses platinum resistance in vivo, underscoring that targeting m^5^C machinery, both writers and readers, represents a promising strategy to overcome chemoresistance [128]. While direct pharmacologic inhibitors of the m^5^C machinery remain limited, advances in targeting other RNA modification enzymes, such as m^6^A and pseudouridine regulators, demonstrate the feasibility of drugging RNA methylation pathways and provide a framework for developing selective m^5^C inhibitors with optimized pharmacokinetic properties [181,186].

#### 7.2.2. Challenges in Specificity and Safety

Despite promising opportunities, therapeutic targeting of m^5^C machinery faces several challenges. First, m^5^C modifications are widespread and context-dependent, raising concerns about off-target effects and unintended disruption of normal RNA metabolism. Many m^5^C writers act on multiple RNA species, including tRNAs, rRNA, and mRNAs, complicating selective intervention. Second, m^5^C plays important roles in normal development, stress responses, and immune regulation, necessitating careful consideration of dose, timing, and tissue specificity to minimize toxicity. A further challenge arises from functional redundancy and overlaps among RNA methyltransferases and reader proteins, which may compensate for inhibition of individual components and limit therapeutic efficacy. Additionally, technical limitations in accurately mapping functional m^5^C sites hinder target prioritization and biomarker development, particularly within complex epitranscriptomic networks where targeting a single modification pathway may produce unpredictable compensatory effects. Advances in high-resolution m^5^C detection, single-cell profiling, and structure-guided drug design will be critical for overcoming these barriers and translating m^5^C-targeted strategies into safe and effective therapies.

## 8. Knowledge Gaps and Future Directions

Despite rapid advances in epitranscriptomics, the understanding of m^5^C RNA modification in women’s health remains incomplete. Addressing current knowledge gaps will be essential for translating descriptive findings into mechanistic insight and clinical application.

### 8.1. Tissue-Specific m^5^C Landscapes in Women

Despite advances in transcriptome-wide m^5^C mapping, most existing studies have relied on bulk tissue samples or cancer cohorts, such as profiling m^5^C methylomes across multiple human tissues or in tumor versus adjacent normal tissues [187,188]. Moreover, reviews of RNA modification research highlight the incomplete characterization of epitranscriptomic marks across diverse cell types and physiological contexts, reflecting a broader gap in tissue-specific, cell–type–specific and sex-stratified analyses [189].

### 8.2. Causality and Functional Validation in m^5^C Epitranscriptomics

A key limitation in current epitranscriptomic studies is their largely correlative nature, making it difficult to distinguish causal roles of m^5^C modifications from secondary transcriptional effects. Although associations between m^5^C regulators and disease phenotypes are well documented, direct mechanistic links remain insufficiently defined. To address this, functional validation is essential. Emerging approaches, including site-specific RNA methylation editing and CRISPR-based manipulation of m^5^C regulatory enzymes, enable direct assessment of m^5^C-dependent effects on RNA stability, localization, and translation. In addition, mapping modification-dependent RNA–protein interactions can help identify key reader proteins and downstream pathways. Integrating these tools with phenotypic assays will be critical to establish causal relationships between m^5^C regulation and disease pathogenesis, thereby strengthening the foundation for therapeutic targeting.

### 8.3. Interaction with Sex Hormones

The interaction between m^5^C regulation and sex hormone signaling remains largely unexplored. Estrogen and progesterone profoundly reshape transcriptional and post-transcriptional programs in female tissues, yet it remains unknown whether these hormones directly regulate m^5^C writers, readers, or target selection. Hormone-dependent modulation of RNA metabolism has been documented, suggesting that m^5^C may function as a downstream effector of steroid hormone signaling. Elucidating these interactions will be critical for understanding hormone-driven diseases such as BC, endometriosis, and UFs.

A major challenge in advancing m^5^C epitranscriptomic research is the lack of preclinical models that accurately reflect human regulation in sex-specific, hormone-dependent contexts. Common in vitro and animal models often fail to capture dynamic hormonal fluctuations, the complexity of receptor signaling, and tissue-specific responses, limiting our ability to assess how estrogen and progesterone influence m^5^C function under physiological conditions. This is particularly relevant as m^5^C-mediated RNA regulation may vary across reproductive states and in hormone-driven diseases. Consequently, the absence of such models hampers accurate evaluation of the safety and efficacy of m^5^C-targeted therapies and may overlook context-dependent effects. Developing advanced systems, including hormone-responsive organoids and patient-derived models, will be essential to better understand m^5^C–hormone interactions and to guide therapeutic strategies for diseases such as BC, endometriosis, and UFs.

### 8.4. Longitudinal and Life-Course Studies

Most m^5^C studies to date provide static snapshots of disease or tissue states. There is a pressing need for longitudinal and life-course–oriented studies that examine m^5^C dynamics across puberty, reproductive years, pregnancy, menopause, and aging. Such approaches are particularly relevant for women’s health, where hormonal transitions and cumulative exposures shape disease risk. Longitudinal profiling could reveal whether m^5^C alterations precede disease onset, reflect adaptive responses, or represent irreversible pathogenic changes.

### 8.5. Single-Cell and Spatial Epitranscriptomics

Bulk analyses obscure cellular heterogeneity that is especially pronounced in reproductive tissues and tumors. Recent single-cell and spatial transcriptomic profiling of the human ovary highlights this complexity: over 18,000 genes were analyzed across 257 spatial regions, and single-cell RNA sequencing of 21,198 cells identified four major cell types and four immune subtypes, revealing distinct gene programs in oocytes, theca, and granulosa cells, as well as spatial variation in hormone and extracellular matrix remodeling activities [87]. These datasets provide a valuable framework for interpreting cell-type–specific functions and molecular programs in the ovary. Applying single-cell and spatial epitranscriptomic technologies to m^5^C could similarly resolve cell-type–specific m^5^C regulation within these complex tissue microenvironments, offering new insights into stromal–epithelial interactions, immune infiltration, and spatial organization in women’s diseases [190,191].

### 8.6. Ethical and Clinical Considerations

As m^5^C profiling moves toward clinical translation, ethical and practical considerations must be addressed. These include data privacy concerns related to RNA-based biomarkers, equitable representation of women across diverse populations, and careful evaluation of unintended consequences when targeting RNA modification pathways that also function in normal physiology. In pregnancy-related contexts, particular caution is warranted to avoid interventions that could adversely affect fetal development. Establishing robust ethical frameworks and clinical guidelines will be essential for the responsible implementation of m^5^C-based diagnostics and therapies.

## 9. Conclusions

This review highlights the expanding role of m^5^C RNA modification as a critical post-transcriptional regulatory layer shaping gene expression across women’s health and disease. Accumulating evidence demonstrates that m^5^C is dynamically regulated by dedicated writer, reader, and eraser proteins and influences RNA stability, translation, cellular stress responses, and inflammatory signaling. Dysregulation of m^5^C machinery has been implicated in hormone-responsive cancers, gynecological and reproductive disorders, metabolic and cardiovascular diseases, and pregnancy-associated pathologies, underscoring the broad physiological relevance of this epitranscriptomic mark.

Importantly, women’s health contexts, characterized by cyclical hormonal fluctuations, reproductive transitions, and pregnancy-related adaptations, may be uniquely sensitive to perturbations in RNA modification pathways. m^5^C emerges as a potential molecular integrator of hormonal signaling, metabolic state, and tissue-specific gene regulation, offering new insights into sex-biased disease susceptibility and progression. Recognizing m^5^C as a contributor to these processes expands our understanding beyond classical epigenetic mechanisms and highlights the need to incorporate epitranscriptomic regulation into models of female biology.

Looking ahead, advances in high-resolution m^5^C mapping, single-cell and spatial epitranscriptomics, and longitudinal study designs will be essential for defining functional m^5^C landscapes across women’s life stages. These efforts, combined with improved detection technologies and mechanistic studies, will accelerate the translation of m^5^C-based biomarkers and therapeutic strategies into clinical practice. Ultimately, integrating m^5^C biology into women’s health research holds promise for more precise diagnostics, personalized therapies, and improved outcomes across a spectrum of female-predominant diseases.

## Figures and Tables

**Table 2:** m^5^C RNA Modifications in Female Cancer.

Diseases	Sample Types	Approaches	Mechanism	Biological Effects	Publication Date	Ref.
Breast cancer	Tumor and adjacent tissues from 5 BC patients; In vitro BC cell models; In vivo BC experimental models	RNA sequencing, RNA-BisSeq (single-based m^5^C mapping), MeRIP-qPCR, RIP-qPCR, proteomics, co-immunoprecipitation, and ribosome profiling.	NSUN2 mediates m^5^C modification of HGH1 mRNA, with YBX1 binding to m^5^C sites to enhance mRNA stability and promote translation via interaction with EEF2.	NSUN2/YBX1-driven m^5^C modification stabilizes HGH1, increases protein synthesis, promotes BC cell proliferation and progression, and represents a potential therapeutic target.	Jun, 2024	[107]
Breast cancer	Human TNBC tumor tissues; TNBC cell lines; mouse xenograft tumor models	RNA interference, lentiviral transduction, in vivo xenografts, ribosome sequencing, tRNA m^5^C bisulfite sequencing, codon usage analysis, glycolysis assays, and molecular docking.	NSUN2 mediates m^5^C modification of tRNA, enhancing codon-biased translation of glycolysis-related genes	Promotes glycolysis, TNBC cell proliferation, migration, invasion, and docetaxel resistance; NSUN2 inhibition suppresses tumor growth and sensitizes cells to chemotherapy	Aug, 2025	[108]
Breast cancer	Human BC cell lines, patient-derived tumor samples	Mapping the m^5^C landscape across different BC subtypes; CRISPR/Cas9 gene editing; Comparative methylation analysis:	NSUN2-mediated m^5^C modification regulates mRNA stability and expression, with subtype-specific hypo- and hypermethylation patterns	Altered m^5^C modifies gene expression networks, contributing to BC progression and providing potential therapeutic targets.	Jun, 2025	[109]
Breast cancer	Human BC cell lines	Biochemical validation of NSUN6 activity on mRNA, site-directed mutagenesis to separate mRNA vs. tRNA catalytic functions, RNA stability assays, binding studies with m^5^C readers (YBX1 and YBX3), and functional assays for cell migration.	NSUN6 catalyzes high-stoichiometry, site-specific m^5^C modification; these m^5^C-modified mRNAs are stabilized via preferential binding by m^5^C readers YBX1 and YBX3, enhancing mRNA stability and promoting BC cell migration	Increased mRNA stability, enhanced BC cell migration, and potential applicability in stabilizing therapeutic mRNAs for clinical use.	Jul, 2025	[110]
Breast cancer	BC cell models	Protein–protein interaction assays; Transcriptional activity; reporter assays; Gene expression analysis, signaling modulation experiments Complex interaction studies	NFIB and YBX1 bind the ESR1–FOXA1 complex, inhibiting ESR1 transactivation; FGFR2 signaling enhances this interaction, further repressing ESR1 target genes	Suppression of ESR1 target gene expression, promotion of estrogen-independent phenotype, and potential contribution to progression of ER-negative BC	Jan, 2018	[111]
Breast Cancer Brain Metastasis	BCBM tissues and matched primary BC tissues, 231-BR cells (brain metastatic BC cell line)	MeRIP-seq to profile circRNA methylation, bioinformatics analysis of differential m^5^C peaks, knockdown of circRNA (hsa_circ_0004516), *NSUN2* depletion and catalytic mutant analysis	NSUN2 catalyzes m^5^C methylation of hsa_circ_0004516, enhancing its stability in a catalytic-dependent manner and thereby activating AKT signaling to establish the NSUN2–m^5^C–circRNA–AKT axis.	Increased proliferation, migration, and invasion of metastatic BC cells, promoted brain metastasis progression	Aug, 2025	[112]
Breast Cancer Brain Metastasis	BCBM tissues and BC cell lines, In vivo brain metastasis models	Gene overexpression and functional assays In vivo metastasis studies, m^5^C epitranscriptomic analysis, mutational analysis (NSUN2 catalytic mutants), Investigation of m^5^C reader involvement	NSUN2-mediated m^5^C methylation of *PSD4* mRNA enhances its stability and nuclear export, with YBX1 binding facilitating PSD4 regulation, leading to ferroptosis resistance and vasculogenic mimicry	This regulation promotes cancer cell proliferation, invasion, migration, EMT, vasculogenic mimicry, reduced vascular permeability, ferroptosis resistance, and ultimately brain metastasis progression.	Nov, 2025	[113]
Breast cancer with bone metastasis	Human bone metastatic lesions vs. adjacent non-metastatic bone tissues; experimental BC cell lines and in vivo mouse models	NSUN2 overexpression and knockdown, m^5^C RNA modification assays, osteoclast differentiation assays	NSUN2- mediates m^5^C on *KDM6B* mRNA promotes its degradation, activates Notch signaling via NUMB hypermethylation upregulating RANKL, thereby accelerating osteoclast differentiation through the NSUN2–KDM6B–Notch axis.	Increased osteoclast differentiation, enhanced osteolytic bone metastasis, and activation of bone-tumor microenvironment signaling; pharmacological targeting of KDM6B or Notch pathway mitigates these effects.	Oct, 2025	[114]
Breast cancer	TNBC patient samples, TNBC cell lines, xenograft mouse model	Expression and clinical correlation, functional studies, protein–protein interaction study	SAT1 stabilizes YBX1 via deubiquitination, enhancing m^5^C-mediated stabilization of mTOR mRNA and suppressing autophagy	Increased proliferation, migration, tumor growth, and autophagy deficiency leading to TNBC progression	Sep, 2024	[115]
Breast Cancer	BC cell line	Epitranscriptomic profiling, functional studies, transcriptomic and network analysis	m^5^C elevation in tamoxifen resistance, NSUN7-mediated m^5^C modification regulates genes	Increased tamoxifen resistance, enhanced migration and clonogenicity, and association with poor prognosis	Dec, 2025	[116]
Breast and Ovarian cancer	Human OC tissues and human BC cell line	Gene expression analysis in the GTEx database and NSUN6 knockdown studies.	NSUN6 is downregulated in OC. Knockdown NSUN6 decreased BC migration.	NSUN6 may exert tumor-suppressive effects in OC but tumor-promoting effects in BC	Jan, 2021	[117]
Ovarian cancer	Human OC transcriptomic datasets	Molecular Subtyping, DEG analysis, Risk Model Construction related to m^5^C-related genes	Dysregulated m^5^C regulatory genes alter RNA methylation and gene expression networks driving tumor progression	Distinct prognostic subtypes, enhanced malignant progression, and independent survival prediction	Jun, 2021	[118]
Ovarian cancer	OC patients from TCGA	Consensus clustering, pathway enrichment analysis, chemotherapy sensitivity prediction, LASSO prognostic modeling	Distinct m^5^C regulatory patterns drive metabolic heterogeneity, influencing tumor progression and chemotherapy response	Differential survival outcomes, varied chemotherapy sensitivity, and prognostic stratification based on m^5^C-related genes	Mar, 2022	[119]
Ovarian Cancer	TCGA OC RNA-seq and clinical dataset	Nonnegative matrix factorization (NMF) clustering, differential expression analysis, LASSO regression modeling, survival and immune correlation analysis	Dysregulation of RNA-modification regulatory genes drives epitranscriptomic heterogeneity affecting tumor biology and immune microenvironment	Distinct prognostic subtypes, altered drug sensitivity, immune modulation, and survival differences	Dec, 2022	[120]
Ovarian cancer	TCGA and ICGC datasets	MeRIP-seq identification of m^5^C genes and LASSO-based prognostic modeling with immune analysis	m^5^C-related genes regulate tumor progression and immune microenvironment	Predicts survival and immunotherapy response in OC patients	Nov, 2023	[121]
Ovarian cancer	Human OC patient tumor datasets/tissues and OC cell models	RNA methylation assays, mRNA stability analysis, transcription factor binding studies, phase separation assays, and gene expression/prognostic correlation analysis	NSUN2 mediates E2F1 mRNA(m^5^C), which is recognized by YBX1 to enhance E2F1 expression, E2F1 then activates NSUN2, forming a positive feedback loop	Upregulation of E2F1, activation of oncogenes MYBL2 and RAD54L, promotion of tumor progression, and association with poorer prognosis in OC	Mar, 2024	[122]
Ovarian cancer	TCGA transcriptomic dataset	Multi-omics bioinformatics clustering and Cox/random forest-based risk modeling	Epigenetic (m^6^A/m^5^C/m^1^A/m^7^G)-related immune lncRNAs regulate tumor microenvironment and mutation heterogeneity	Defines prognostic subtypes and predicts immune checkpoint and chemotherapy response	Aug, 2023	[123]
Ovarian cancer	HGSOC tissue	m^5^C profiling of lncRNAs, RNA-seq integration, pathway enrichment, clinical correlation analysis	Dysregulated m^5^C methylation in lncRNAs alters PI3K-Akt and cancer-related signaling pathways	Increased tumor growth, invasion, metastasis, and poor prognosis	Dec, 2021	[124]
Ovarian cancer	HGSOC and normal fimbria tissues	Expression analysis, functional characterization, downstream target identification, and m^5^C RNA methylation analysis.	NSUN1 overexpression may increase m^5^C methylation, enhance RAPGEF4 expression, and subsequently activate pro-tumorigenic signaling pathways.	NSUN1 is involved in cell proliferation, migration, and invasion, and may serve as a potential diagnostic biomarker.	Dec, 2023	[125]
Ovarian cancer	OC cell lines (OVCAR3, SKOV3) and in vivo models	Sevoflurane treatment with DNMT2/APC modulation and functional assays for malignancy and m^5^C methylation	DNMT2-mediated m^5^C modification of APC mRNA activates β-catenin signaling under Sevoflurane exposure	Increased proliferation, migration, invasion, cell cycle progression, reduced apoptosis, and enhanced tumor growth	Dec, 2024	[126]
Ovarian cancer	OC tissues, cell lines, and xenografts	m^5^C binding studies, functional cell assays, and in vivo tumor models	ALYREF stabilizes m^5^C-modified BIRC5 mRNA to drive oncogenic processes	Promotes OC proliferation, migration, invasion, glycolysis, and tumor growth; knockdown inhibits malignant progression	Aug, 2025	[127]
Ovarian cancer	PDOs, OC cells, and xenograft models	m^5^C–YBX1 interaction analysis and functional drug-resistance assays	YBX1 binds m^5^C-modified CHD3 mRNA to enhance homologous recombination and DNA repair	Promotes platinum resistance and tumor survival under chemotherapeutic stress	Aug, 2024	[128]
Ovarian cancer	Cisplatin-resistant OC cell lines and patient samples	Multi-omics profiling and functional ALYREF/NSUN2 modulation assays	NSUN2-mediated m^5^C on LGR4 mRNA is recognized by ALYREF, stabilizing LGR4 and activating Wnt/β-catenin signaling	Promotes chemoresistance and survival of OC cells	Dec, 2025	[129]
Ovarian cancer	OC tissues, cell lines, and PDX models	m^5^C profiling, YBX1/MATR3 functional assays, and in vitro/in vivo chemosensitivity testing	YBX1 stabilizes m^5^C-modified CCNL2 mRNA, cooperating with MATR3 to promote proliferation and chemoresistance	Enhanced tumor growth and cisplatin resistance; YBX1 inhibition restores chemosensitivity	Jul, 2025	[130]
Ovarian cancer	Chemoresistant and chemosensitive tissues, epithelial ovarian cancer (EOC) cell lines, xenograft models	Expression analysis, ubiquitination assays, functional overexpression studies, in vivo validation	SIAH1 ubiquitinates YBX1 at Lys304, promoting its degradation and destabilizing m^5^C-modified mRNAs	Increased cisplatin sensitivity, reduced tumor growth, and reversal of chemoresistance	Mar, 2022	[131]
Ovarian cancer	OC patient samples from databases and in vitro OC cell lines	Identification of m^5^C-related lncRNAs, nine-lncRNA risk model via LASSO-Cox regression, survival and nomogram analysis, GSEA, immune infiltration, WGCNA, ceRNA network, and functional assays	m^5^C-related lncRNAs regulate gene expression and immune infiltration, promoting tumor progression and OC cell proliferation	Risk model stratifies prognosis; AC005562.1 drives OC cell proliferation; pathways and immune profiles differ by risk group; potential biomarkers identified	May, 2023	[132]
Ovarian cancer	OC cell lines and tumor-bearing mice	m^5^C-modification analysis, lncRNA/miRNA interaction studies, functional and in vivo assays	NSUN2-mediated m^5^C stabilizes SNHG15, which sponges miR-545-3p to upregulate PD-L1	Promote proliferation, immune evasion, and tumor growth; knockdown of SNHG15 restores anti-tumor immunity	Aug, 2025	[133]
Uterine corpus endometrial carcinoma (UCEC)	TCGA transcriptomic dataset	Consensus clustering and LASSO-based prognostic risk modeling with immune infiltration analysis	m^5^C-related lncRNAs influence tumor progression and immune microenvironment	Predicts prognosis and correlates with tumor grade, PD-L1 expression, and immune infiltration	Mar, 2022	[134]
Endometrial cancer	EC patient tissues, EC cell lines, and in vivo tumor xenograft models	Epigenetic and m^5^C sequencing analyses, ferroptosis assays, knockdown/rescue experiments	NSUN2-mediated m^5^C stabilizes SLC7A11 via YBX1, suppressing ferroptosis	Promotes proliferation and ferroptosis resistance; targeting NSUN2 enhances ferroptotic tumor suppression	Feb, 2024	[135]
Endometrial cancer	TCGA/GEO datasets and in vitro cell experiments	Clustering, pathway enrichment, and LASSO-based prognostic modeling	m^5^C regulators (NSUN2, YBX1) modulate cell adhesion pathways	Distinct m^5^C patterns correlate with prognosis and survival prediction	Oct, 2023	[136]
Cervical cancer	CC cancer cell line	Transcriptome-wide m^5^C profiling combined with NSUN2 depletion/rescue, RNA-binding assays, and mRNA export analyses.	NSUN2 installs m^5^C on CG-rich mRNA regions, which are recognized by ALYREF to facilitate nuclear–cytoplasmic mRNA export.	m^5^C promotes efficient mRNA export and regulates post-transcriptional gene expression in mammalian cells.	Apr, 2017	[18]
Cervical, ovarian, and endometrial cancer	Gynecologic cancer cell lines (cervical, ovarian, endometrial); comparative cellular models across cancer types	NSUN2 knockdown and overexpression, migration and invasion assays, catalytic activity rescue experiments, and molecular interaction analysis.	NSUN2 mediates m^5^C methylation of KRT13 mRNA, which is recognized and stabilized by the m^5^C reader YBX1, promoting oncogenic activity.	In CC, NSUN2 depletion inhibits cell migration and invasion, whereas NSUN2 has minimal functional impact in ovarian and EC cells, highlighting a cancer-specific oncogenic pathway	Feb, 2022	[137]
Cervical cancer	CC tissues, cell lines, and in vivo models	Functional assays, m^5^C modification analysis, RNA-protein interaction studies	NSUN2-mediated m^5^C stabilizes LRRC8A via YBX1 binding	Promotes proliferation and metastasis by suppressing apoptosis	Jan, 2023	[138]
Cervical cancer	Clinical CC specimens and CC cell lines (Caski, SiHa)	Bioinformatics, proliferation/migration assays, Western blot, knockdown/rescue, m^5^C RNA analysis	NSUN2-mediated m^5^C stabilizes LINC01094, which activates SIRT1 via ZNF582, suppressing p53	Promotes EMT, metastasis, proliferation, and inhibits apoptosis in CC cells	Feb, 2026	[139]
Cervical cancer	Patient samples, cell lines, CDX, and 3D PDO models	LC-MS/MS, RNA-seq, m^5^C-seq, RIP, functional radiosensitivity assays	NSUN6-mediated m^5^C modification stabilizes NDRG1 mRNA via ALYREF, enhancing homologous recombination DNA repair	Promotes radioresistance and poor prognosis; NSUN6 inhibition restores radiosensitivity	Jul, 2024	[140]
Cervical cancer	CC cell lines	MeRIP, RIP, Co-IP, RT-qPCR, Western blot, functional ferroptosis and viability assays	ALYREF stabilizes KIF20A mRNA via m^5^C methylation; KIF20A activates BUB1 to induce ferroptosis resistance	Promotes hypoxia-induced ferroptosis resistance; disruption of the ALYREF/KIF20A/BUB1 axis sensitizes cells to ferroptosis	Jan, 2026	[141]
Cervical cancer	Tumor tissues	m^5^C mapping, transcriptomics, knockdown/overexpression, proliferation, metastasis, and drug sensitivity assays	m^5^C stabilizes SERPINB5 mRNA, promoting mitotic regulators (CENPE) to overcome drug-induced mitotic arrest	Drives tumor growth, metastasis, and chemoresistance; SERPINB5 depletion restores sensitivity to paclitaxel and vincristine	Feb, 2026	[142]
Cervical cancer	TCGA/GEO datasets, CC tissues, SiHa cells	NMF clustering of m^5^C regulators, Cox-based 4-gene signature construction, experimental validation	m^5^C modification subtypes drive differential expression of oncogenic genes (FNDC3A, VEGFA, OPN3, CPE)	Defines prognostic subtypes; high-risk signature correlates with poor survival and enhanced proliferation and invasion	Sep, 2021	[143]
Cervical cancer	Clinical samples and public datasets (TCGA, GSE39001)	Bioinformatics modeling, COX regression, immune infiltration analysis, IHC/RT-qPCR validation, and drug screening	m^6^A, m^5^C, and m^1^A methylation-related genes regulate tumor progression and immune microenvironment	Predicts survival, correlates with immune infiltration, and identifies potential immunotherapy targets	Dec, 2023	[144]
Cervical cancer	Clinical samples from TCGA database	Bioinformatics-based lncRNA-methylation gene signature construction and validation with survival, immune, and drug response analyses	m^1^A/m^6^A/m^5^C-associated lncRNAs regulate gene expression and immune pathways	Predicts prognosis, immunotherapy response, and potential drug sensitivity	Jan, 2024	[145]

**Table 3:** The Role of m^5^C RNA Modifications in Cardiovascular Disease.

Disease	Sample Type	Approaches	Mechanism	Biological Effects	Publication Date	Ref.
Mitochondrial dysfunction–associated cardiomyopathy	Conditional *Nsun4* knockout mouse model; mouse mitochondrial RNA; mitochondrial ribosomal fractions	Conditional knockout; mitochondrial translation assays; bisulfite RNA sequencing; ribosome assembly and NSUN4–MTERF4 interaction analysis; mitoribosome biogenesis assays	NSUN4 independently methylates C911 (m^5^C911) in mitochondrial 12S rRNA, while the NSUN4–MTERF4 complex promotes assembly of ribosomal subunits into a functional monosome	Loss of *Nsun4* abolishes mitochondrial translation and disrupts ribosome maturation, impairing mitochondrial protein synthesis and cellular energy homeostasis	Feb, 2014	[14]
Vascular inflammation/allograft arteriosclerosis	Cultured vascular endothelial cells; wild-type rat vascular tissue; *Nsun*−/− rat vascular tissue; rat aortic allograft specimens	In vitro and in vivo mRNA methylation assays, leukocyte adhesion assays, TNF-α and homocysteine stimulation, *Nsun2* knockout models, and allograft arteriosclerosis assessment	NSUN2-driven m^5^C methylation amplifies inflammatory endothelial signaling via translational control of ICAM-1	*Nsun2* deficiency reduces ICAM-1 induction and leukocyte adhesion attenuating vascular inflammation	Mar, 2016	[148]
Cardiac hypertrophy	Dnmt2-deficient mouse model; mouse cardiac tissue; isolated cardiomyocytes	Genetic knockout mouse model; echocardiography; cardiac morphometric analysis; cellular hypertrophy assays; molecular analysis of P-TEFb complex activity; noncoding RNA interaction studies	Dnmt2 deficiency promotes dissociation of the inhibitory noncoding RNA 7SK from the P-TEFb complex, resulting in increased P-TEFb activation and transcriptional programs that drive cardiac growth	Loss of *Dnmt2* leads to cardiomyocyte enlargement and cardiac hypertrophy while preserving systolic function	Jun, 2016	[149]
Abdominal aortic aneurysm (AAA)	Human AAA tissue (*n* = 32) vs. normal aortic tissue (*n* = 12)	Global m^5^C methylation quantification; gene expression analysis; m^5^C regulators; protein expression analysis; (RIP-seq; Regulatory network analysis	m^5^C dysregulation enhances immune-related lncRNA–mRNA networks, promoting inflammatory signaling and macrophage infiltration in AAA tissue	Elevated m^5^C and its regulators are associated with inflammatory cell infiltration and immune activation, contributing to AAA pathogenesis	Nov, 2021	[150]
Cardiac regeneration	Human iPSCs; hiPSC-derived cardiomyocytes; metabolically matured CMs; biochemically enhanced CMs.	Transcriptome-wide RNA-BS-seq integrated with RNA-seq to profile differential m^5^C methylation and gene expression during cardiomyocyte differentiation and maturation.	Dynamic remodeling of RNA m^5^C during pluripotency exit and cardiac lineage commitment modulates lineage-specific transcript regulation.	Biological effect: m^5^C reprogramming supports cardiomyocyte differentiation and maturation by regulating stem cell pluripotency and cardiac gene expression programs	Dec, 2024	[157]
Atherosclerosis/vascular restenosis	Human aortic vascular smooth muscle cells	nucleolar morphology analysis, oxidative DNA damage assays, protein expression (p53, p21), rDNA transcription assessment, SIRT7 and DNMT2 expression, RNA methylation analysis	Curcumin downregulates SIRT7 and upregulates DNMT2, inhibiting rDNA transcription and promoting RNA methylation, leading to nucleolar stress and cell cycle arrest	Oxidation-dependent cytostatic effect in VSMCs, reducing proliferation and potentially limiting atherosclerosis or post-angioplasty restenosis.	Mar, 2015	[158]
Pressure overload–induced myocardial remodeling/heart failure	TAC-induced hypertrophic mice; angiotensin II–treated H9c2 cells and neonatal rat cardiomyocytes (NRCMs).	Echocardiography, histological staining, Western blot, Dot blot and IHC for m^5^C, RNA-seq, molecular docking, MST, NSUN2 knockdown, and MeRIP-qPCR validation.	Puerarin upregulates and targets NSUN2, enhancing m^5^C methylation of NF-κB mRNA, which promotes its degradation and suppresses NF-κB signaling activation.	Increased NSUN2-mediated m^5^C modification reduces cardiomyocyte hypertrophy and fibrosis, improves cardiac function, and attenuates myocardial remodeling progression	Jul, 2025	[161]
Ischemia/reperfusion–induced cardiomyocyte necroptosis/ischemic heart injury	Hypoxia/reoxygenation (H/R)–treated cardiomyocytes; I/R-injured mouse hearts	piRNA expression analysis, HNEAP knockdown, cardiac function assessment in mice, RNA–protein interaction assays, m^5^C methylation analysis, and Atf7/Chmp2a expression studies	HNEAP binds DNMT1, suppressing m^5^C methylation of Atf7 mRNA, elevating ATF7 levels, which represses Chmp2a transcription and promotes cardiomyocyte necroptosis	HNEAP promotes necroptotic cardiomyocyte death and worsens cardiac function, while its inhibition reduces necroptosis and protects the heart	Dec, 2023	[162]
Sepsis-induced myocardial injury (SIMI)	Cecum ligation and puncture (CLP) rat model of SIMI; LPS-treated H9c2 cardiomyocytes in vitro.	RT-qPCR, ELISA, ferroptosis and Western blot assays, MeRIP-qPCR and RIP, luciferase reporter assays, and in vivo/in vitro NSUN2 or NCOA4 overexpression	NSUN2 mediates m^5^C modification of NCOA4 mRNA, suppressing its expression and thereby inhibiting NCOA4-driven ferroptosis and inflammatory signaling.	NSUN2 overexpression reduces inflammation, ferroptosis, myocardial injury, and cardiac dysfunction in SIMI, while improving cardiomyocyte viability	Jul, 2025	[163]
Doxorubicin-induced cardiotoxicity (DIC)	DOX-treated murine model; cardiomyocyte-specific ALYREF overexpression and knockout mice; cultured cardiomyocytes	Cardiac-specific genetic manipulation (overexpression and CKO), DNA damage and apoptosis assays, protein interaction and binding analysis, phase separation (LLPS) assays, and ubiquitination studies	DOX binds ALYREF, disrupts its liquid–liquid phase separation, promotes ubiquitin-mediated degradation, destabilizes the NORAD-activated ribonucleoprotein complex, and impairs genomic integrity	Loss of ALYREF condensates enhances cardiomyocyte DNA damage, apoptosis, and cardiac dysfunction, whereas preserving ALYREF phase separation mitigates DOX-induced cardiotoxicity	Nov, 2025	[164]
Post-myocardial infarction cardiac fibrosis	Myocardial ischemia mouse model with myofibroblast-specific NSUN2 knockdown; cardiac myofibroblasts.	Conditional myofibroblast-specific *Nsun2* knockdown, fibrosis assessment, and RNA stability evaluation of HuR mediated by m^5^C modification.	NSUN2-mediated m^5^C modification enhances HuR mRNA stability, promoting pro-fibrotic gene expression in myofibroblasts	Myofibroblast-specific *Nsun2* knockdown reduces HuR stability and suppresses extracellular matrix deposition, thereby attenuating cardiac fibrosis after myocardial ischemia	Dec, 2025	[165]
Doxorubicin-induced cardiotoxicity	C57BL/6J mice treated with DOX; H9C2 cardiomyocytes in vitro	*NSUN2* knockdown or overexpression, cardiac function assessment, histopathology, apoptosis and ROS assays, NRF2 protein/mRNA analysis, and m^5^C MeRIP assays	NSUN2 methylates *Nrf2* mRNA (m^5^C), increasing its stability and protein expression, which enhances the antioxidative stress response	NSUN2 protects cardiomyocytes from DOX-induced apoptosis and oxidative damage, preserving cardiac function	Feb, 2023	[166]
Cardiac hypertrophy/heart failure	Human hypertrophic heart tissues; neonatal rat cardiomyocytes; adult mouse cardiomyocytes; cardiac-specific Nsun2 knockout mice under aging, transverse aortic constriction (TAC), and angiotensin II stress.	Bulk and single-cell RNA-seq, m^5^C-RIP-seq, Cre/LoxP cardiac-specific knockout, rAAV9-mediated gene modulation, RNA pull-down, polysome profiling, reporter assays, and IonOptix contractility measurements.	NSUN2 catalyzes m^5^C methylation of PRKACA mRNA, enhancing its translation via YBX1-dependent regulation and promoting activation of PKA signaling.	NSUN2-driven PRKACA translation sustains cardiac homeostasis and hypertrophic adaptation, whereas Nsun2 loss impairs PKA signaling, calcium handling, contractility, and accelerates heart failure progression.	Jan, 2025	[167]
Hypoxic pulmonary hypertension	Hypoxia-induced PH models and pulmonary artery smooth muscle cells (PASMCs)	circRNA expression analysis, m^5^C prediction and validation, NSUN2 knockdown, circRNA overexpression, RNA–protein interaction assays (FXR2 binding), and PASMC proliferation assays under hypoxia.	NSUN2-mediated m^5^C modification stabilizes circCCNL2, enabling it to bind and sequester FXR2, thereby preventing FXR2 from interacting with CDKL3 and limiting pro-proliferative signaling in PASMCs.	m^5^C-modified circCCNL2 suppresses PASMC proliferation and attenuates pulmonary vascular remodeling, thereby slowing hypoxic PH progression.	Mar, 2025	[168]
Atherosclerosis (AS)	Public GEO microarray datasets; THP-1–derived macrophages/foam cell model; single-cell RNA-seq immune profiling data.	DE/correlation analysis, PPI, LASSO-ROC modeling, clustering, WGCNA, scRNA-seq, qRT-PCR validation, NSUN3 functional assays, and ceRNA prediction	m^5^C regulators (NSUN3/NSUN5) correlate with immune infiltration, and NSUN3 enhances macrophage inflammatory cytokine production	Five diagnostic biomarkers were identified, and NSUN3 drives proinflammatory macrophage responses that promote atherosclerosis progression	Jan, 2026	[169]
Myocardial infarction–induced cardiac remodeling	Mouse left anterior descending (LAD) ligation MI model; infarcted mouse myocardial tissue; hypoxia-induced primary neonatal cardiac fibroblasts; single-cell sequencing datasets	In vivo AAV-mediated *Alyref* knockdown, siRNA silencing in fibroblasts, single-cell RNA-seq analysis, RIP-seq, immunofluorescence, histopathology, Western blot, and ECM gene expression assays.	The m^5^C reader ALYREF binds target transcripts to enhance extracellular matrix protein synthesis (Col1a2, Col3a1, Eln) via regulation of the Fbln1/Loxl1 pathway in activated cardiac fibroblasts	ALYREF promotes fibroblast proliferation, transdifferentiation, and collagen/elastin deposition, thereby facilitating post-MI cardiac remodeling, while its silencing impairs ECM synthesis and worsens cardiac function	Jun, 2025	[170]
Acute coronary syndrome (ACS)	Serum from ACS patients; ox-LDL–stimulated cardiac microvascular endothelial cells (CMECs).	siRNA TET3 knockdown, RT-qPCR/Western blot, proliferation–apoptosis assays, m^5^C and RNA stability analysis, RIP, and CCAT2/TRIM14 functional studies	TET3 removes m^5^C modification from lncRNA CCAT2, reducing its stability and weakening CCAT2–FUS interaction, which releases TRIM14 mRNA from repression and increases TRIM14 expression.	Elevated TET3 promotes CMEC apoptosis and impairs proliferation and angiogenesis, thereby aggravating endothelial damage in ACS	Nov, 2025	[171]

## Data Availability

No new data were created or analyzed in this study.

## Abbreviations

AAA	Abdominal aortic aneurysm
ALKBH	AlkB homolog family
ALYREF	Aly/REF export factor
AUC	Area under the curve
BC	Breast cancer
ca^5^C	5-carboxylcytidine
CEA	Carcinoembryonic antigen
CC	Cervical cancer
cfRNA	Cell-free RNA
CVD	Cardiovascular disease
DNMT2/TRDMT1	DNA methyltransferase 2/tRNA aspartic acid methyltransferase 1
EOC	Epithelial ovarian cancer
EVs	Extracellular vesicles
f^5^C	5-formylcytidine
GDM	Gestational diabetes mellitus
HGSOC	High-grade serous ovarian cancer
hm^5^C	5-hydroxymethylcytidine
HPV	Human papillomavirus
ICAM-1	Intercellular adhesion molecule 1
KGN	Human granulosa-like tumor cell line
lncRNA	Long noncoding RNA
LPS	Lipopolysaccharide
m^1^A	N1-methyladenosine
m^5^C	5-methylcytidine
m^6^A	N^6^-methyladenosine
miRNA	MicroRNA
mRNA	Messenger RNA
MST	Microscale thermophoresis
MZT	Maternal-to-zygotic transition
ncRNA	Noncoding RNA
NLRP3	NLR family pyrin domain containing 3
NMF	Nonnegative matrix factorization
NSUN	NOP2/Sun RNA methyltransferase family
PCOS	Polycystic ovary syndrome
P-TEFb	Positive transcription elongation factor b
RNA-BS-Seq	RNA bisulfite sequencing
RIP-seq	RNA immunoprecipitation sequencing
ROC	Receiver operating characteristic
rRNA	Ribosomal RNA
SAM	S-adenosylmethionine
TNBC	Triple-negative breast cancer
tRNA	Transfer RNA
TET	Ten-Eleven Translocation family
TNF-α	Tumor necrosis factor alpha
TREX	Transcription–Export complex
UTR	Untranslated region
YBX1	Y-box binding protein 1
YTHDF2	YTH N^6^-methyladenosine RNA binding protein F2
ZGA	Zygotic genome activation
Ψ	Pseudouridine
α-KG	Alpha-ketoglutarate
