# Correction: Yang et al. 5-Methylcytidine RNA Epitranscriptomics in Women’s Health and Disease: Mechanisms and Clinical Implications. *Cells* 2026, *15,* 847

**DOI:** 10.3390/cells15131201

**Published:** 2026-07-02

**Authors:** Qiwei Yang, Sana M. Salih, Rongxue Wu, Itika Arora, Mira Mousa, Ayman Al-Hendy, Thomas G. Boyer

**Affiliations:** 1 Department of Obstetrics and Gynecology, University of Chicago, Chicago, IL 60637, USA; aalhendy@bsd.uchicago.edu; 2 Division of Reproductive Endocrinology and Infertility, Department of Obstetrics and Gynecology, University of Chicago, Chicago, IL 60637, USA; sana.salih@bsd.uchicago.edu; 3 Section of Cardiology, Department of Medicine, Biological Sciences Division, University of Chicago, Chicago, IL 60637, USA; 4 Division of Cardiac Surgery, Department of Surgery, Dorothy M. Davis Heart & Lung Research Institute, Ohio State University, Columbus, OH 43210, USA; wu170@osumc.edu; 5 Department of Public Health and Epidemiology, Khalifa University, Abu Dhabi P.O. Box 127788, United Arab Emirates; itika.arora@ku.ac.ae (I.A.); mira.imousa@ku.ac.ae (M.M.); 6 Center for Biotechnology, Khalifa University, Abu Dhabi P.O. Box 127788, United Arab Emirates; 7 Department of Medical Sciences, Khalifa University, Abu Dhabi P.O. Box 127788, United Arab Emirates; 8 Department of Obstetrics and Gynecology, Sheik Shakhbout Medical City, Abu Dhabi P.O. Box 127788, United Arab Emirates; 9 Department of Molecular Medicine, Institute of Biotechnology, University of Texas Health Science Center at San Antonio, San Antonio, TX 78229, USA; boyer@uthscsa.edu

In the original publication [1], there was an error in Figure 3. The left panel showed pseudouridine located only in the 3′UTR. According to the original report, pseudouridine is presented in the 5′UTR, coding region, and 3′UTR. The corrected Figure 3 is shown below. The authors state that the scientific conclusions are unaffected. This correction was approved by the Academic Editor. The original publication has also been updated.

## Figures and Tables

**Figure 3:**
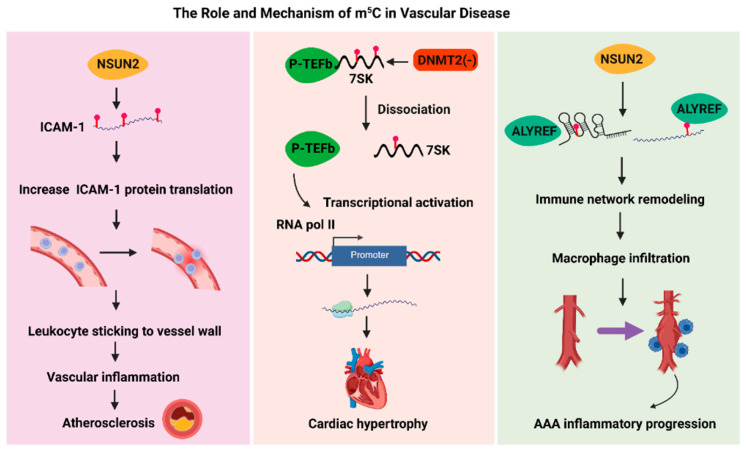
**Epitranscriptomic regulation of cardiovascular pathology by m^5^C RNA methylation.** Left panel: In vascular endothelial cells, the RNA methyltransferase NSUN2 deposits m^5^C modifications on ICAM-1 mRNA, enhancing its translational efficiency. Elevated ICAM-1 expression strengthens leukocyte adhesion to the endothelial surface, initiating a pro-inflammatory vascular niche that promotes sustained immune cell recruitment. This inflammatory amplification contributes to endothelial dysfunction and accelerates atherosclerotic lesion development [148]. Middle panel: In the myocardium, DNMT2 acts as a regulatory brake on transcriptional activation through stabilization of the inhibitory 7SK–P-TEFb complex. Loss of DNMT2 decreases 7SK m^5^C, and promotes dissociation of 7SK, resulting in hyperactivation of P-TEFb–dependent transcriptional programs that drive cardiomyocyte growth. This epitranscriptomic deregulation leads to maladaptive cardiac hypertrophy in the mouse model [149]. Right panel: In abdominal aortic aneurysm (AAA), coordinated upregulation of m^5^C regulators (NSUN2, NSUN5) and the reader protein ALYREF reshapes immune-associated RNA regulatory networks. Aberrant m^5^C signaling enhances macrophage infiltration and inflammatory remodeling of the aortic wall, establishing a self-reinforcing inflammatory microenvironment that accelerates aneurysm progression [150].
